# Improving the accuracy of high-throughput protein-protein affinity prediction may require better training data

**DOI:** 10.1186/s12859-017-1533-z

**Published:** 2017-03-23

**Authors:** Raquel Dias, Bryan Kolaczkowski

**Affiliations:** Department of Microbiology and Cell Science, University of Florida, Gainesville, FL USA

**Keywords:** Protein-protein, Binding affinity, Machine learning, Intermolecular interactions, Scoring functions

## Abstract

**Background:**

One goal of structural biology is to understand how a protein’s 3-dimensional conformation determines its capacity to interact with potential ligands. In the case of small chemical ligands, deconstructing a static protein-ligand complex into its constituent atom-atom interactions is typically sufficient to rapidly predict ligand affinity with high accuracy (>70% correlation between predicted and experimentally-determined affinity), a fact that is exploited to support structure-based drug design. We recently found that protein-DNA/RNA affinity can also be predicted with high accuracy using extensions of existing techniques, but protein-protein affinity could not be predicted with >60% correlation, even when the protein-protein complex was available.

**Methods:**

X-ray and NMR structures of protein-protein complexes, their associated binding affinities and experimental conditions were obtained from different binding affinity and structural databases. Statistical models were implemented using a generalized linear model framework, including the experimental conditions as new model features. We evaluated the potential for new features to improve affinity prediction models by calculating the Pearson correlation between predicted and experimental binding affinities on the training and test data after model fitting and after cross-validation. Differences in accuracy were assessed using two-sample *t* test and nonparametric Mann–Whitney *U* test.

**Results:**

Here we evaluate a range of potential factors that may interfere with accurate protein-protein affinity prediction. We find that X-ray crystal resolution has the strongest single effect on protein-protein affinity prediction. Limiting our analyses to only high-resolution complexes (≤2.5 Å) increased the correlation between predicted and experimental affinity from 54 to 68% (*p* = 4.32x10^−3^). In addition, incorporating information on the experimental conditions under which affinities were measured (pH, temperature and binding assay) had significant effects on prediction accuracy. We also highlight a number of potential errors in large structure-affinity databases, which could affect both model training and accuracy assessment.

**Conclusions:**

The results suggest that the accuracy of statistical models for protein-protein affinity prediction may be limited by the information present in databases used to train new models. Improving our capacity to integrate large-scale structural and functional information may be required to substantively advance our understanding of the general principles by which a protein’s structure determines its function.

**Electronic supplementary material:**

The online version of this article (doi:10.1186/s12859-017-1533-z) contains supplementary material, which is available to authorized users.

## Background

Proteins are involved in the majority of chemical reactions that take place within living cells, making them essential for all aspects of cellular function. Proteins never work in isolation; their functional repertoire is determined by how they interact with various small-molecule, DNA/RNA, protein or other ligands. Ligand affinity is largely determined by a protein’s 3-dimensional structure, which determines the spatial conformation of attractive and repulsive forces between the protein and a potential ligand [1–3]. The affinity with which a protein interacts with various ligands–typically expressed as the dissociation constant (Kd or pKd = −log Kd)—provides critical information about protein function and biochemistry, and has been used for the discovery and optimization of novel pharmaceuticals [4–6].

High-throughput prediction of protein-ligand affinity is typically conducted using a fast statistical “scoring function” that decomposes binding affinity into component atom-atom interaction terms representing the attractive and repulsive forces acting across the protein-ligand complex [7, 8]. Although scoring functions can be derived directly from physical chemistry principles [9], the most effective approaches are usually “trained” using large databases of structural complexes with associated experimentally-determined binding affinities [10–12]. After training, a model’s expected predictive accuracy can be gauged by correlating its predicted affinities with experimentally-determined values across a novel dataset not included in training [13, 14].

Many scoring functions are capable of using only the atomic interactions extracted from crystal structures to rapidly predict protein-small molecule affinities with >70% correlation, which is commonly considered adequate to support structure-based drug design [11, 15–21]. Recently, we developed efficient statistical models capable of predicting protein-DNA/RNA affinities with similar accuracy [22]. Our structure-based prediction models also revealed that different combinations of atom-atom interactions are important for predicting different types of protein-ligand complexes. However, no statistical models we examined were capable of predicting protein-protein affinity with >60% correlation, even under the ‘best case’ scenario in which the protein-protein complex was known experimentally.

Accurate prediction of protein-protein interactions is a major goal of computational structural biology, and many approaches have been examined to improve the accuracy of protein-protein affinity prediction [23]. The structural basis of protein-protein interactions is typically more complex and flexible than other protein-ligand interactions, suggesting that entropic forces may be more important in protein-protein interactions [24, 25]. Physics-based approaches such as molecular dynamics can model entropic factors and produce highly-accurate affinity predictions but are too computationally complex to support high-throughput analyses [10–12, 17, 26, 27]. As an alternative approach, smaller manually-curated affinity benchmarks have been proposed to improve the accuracy of high-throughput statistical affinity prediction [28]. However, predictive accuracy on manually-curated datasets rarely exceeds ~60% correlation [29], and accuracy achieved using carefully curated datasets may not generalize well to new data. Importantly, the specific factors that may influence statistical prediction of protein-protein affinity have not been identified, making it difficult to devise reasonable strategies to improve current methods.

## Methods

### Structural dataset curation

X-ray and NMR structures of protein-protein complexes and their associated binding affinities (−log_10_-transformed dissociation constants, pKds) were obtained from PDBbind [30] and from the protein-protein affinity benchmark database [28]. Complexes with ambiguous ligand information were excluded, as were complexes with multiple ligands or mulitimeric proteins, similar to previous approaches applied for building refined protein-ligand data sets [11, 30, 31]. From each protein − protein complex, we extracted a suite of non-redundant atom-atom interactions thought to potentially correlate with ligand affinity. Details on how each atom-atom interaction is defined and calculated are presented in our previous work [22]. We included only those atomic interactions that could be determined entirely from atomic coordinates and atom types in a standard PDB file.

For each protein-protein complex, we extracted additional information on structure acquisition method, temperature, pH, and crystal resolution from the Protein Data Bank [32]. We constructed data sets of 569 protein-protein complexes with assigned temperature data, 545 complexes with pH information and 622 complexes with acquisition method and resolution information. When several temperature values were available for the same structure, we used the mean temperature. We constructed filtered data sets based on structural resolution and acquisition method, with 205 high-resolution structures (≤2.5 Å) and 165 NMR structures.

For each complex, we extracted additional information on binding assay pH, temperature, and methodology from the protein-protein affinity benchmark database, which is a nonredundant set of 144 protein-protein complexes with detailed information on the experimental methods used for measuring binding affinities [28]. We extracted pH data for 127 complexes, temperature data for 103 complexes and binding assay technology for 136 complexes. Information available for each protein-protein complex is provided in Additional file 1: Table S1.

### Statistical modeling, model selection and cross-validation

We updated the original version of our protein-protein affinity prediction model [22] by adding parameters for estimating hydrophobic surface tension and hydrophobicity. The hydrophobicity algorithm used is adapted from [33]. Each amino acid in the surface has a pre-defined hydrophobicity score, modulated by peptide endings and varying between approximately −1 (most hydrophilic) and +2 (most hydrophobic). The surface tension parameter was calculated by summing the atomic contributions of each amino acid to the protein surface tension. These atomic contribution scores were adapted from [34]. Other atom-atom interaction terms in the present statistical model are identical to those defined and evaluated in our previous work [22].

Statistical models were implemented using a generalized linear model framework (GLM, implemented in the GLMULTI package in R), assuming a Gaussian error distribution with logarithmic link function. We used the GLMULTI genetic algorithm to generate 500 candidate models (default parameters, except population size = 500, level = 2, and marginality enabled) and selected the best-fit model for each training dataset using the Akaike information criterion (AIC).

We evaluated the potential for new features to improve affinity prediction models by calculating the Pearson correlation (*r* ^2^) between predicted and experimental binding affinities on the training data after model fitting, which represents the ‘best-case’ possible accuracy.

We then used cross-validation to estimate the expected accuracy of each trained statistical model when applied to new data and to evaluate possible model over-fitting to training data [13, 14]. For each model, we performed 100 replicates of leave-one-out cross-validation. For each replicate, we randomly partitioned the structural data into a testing data set of size *n* = 1, with the remaining complexes used to train the regression model. We calculated the Pearson correlation (*r* ^2^) and root mean squared deviation (RMSD) between predicted and experimentally-determined binding affinities on the unseen testing data and report the average *r* ^2^ and RMSD of each model over the 100 replicates.

Differences in accuracy were assessed using the parametric two-tailed, two-sample *t* test, assuming unequal variances, and the nonparametric Mann–Whitney *U* test. For evaluating the effects of dataset subsampling on predictive accuracy, we used Fisher’s z-transformation, which incorporates a correction for comparing results obtained on a subsample to results from the full dataset (33). In addition, we performed 1000 replicates of random subsampling to evaluate the expected effect of subsampling on predictive accuracy.

## Results

Statistical prediction of protein-protein binding affinity relies on information extracted from large structure-affinity databases [29, 35]. Accuracy and generalizability of predictive models is therefore expected to depend on the quantity and quality of information in the training database as well as the particular types of information available [36]. To evaluate how various aspects of structure-affinity databases affect the accuracy of protein-protein affinity prediction, we examined 1577 protein-protein complexes from the PDBbind database, a comprehensive collection of experimentally-determined affinity measurements assigned to 3-dimensional structural complexes, commonly used to evaluate affinity prediction algorithms [30].

We found that nearly 2/3 of the protein-protein complexes in PDBbind had ambiguous affinity measurements or multiple ligands, making it difficult to confidently assign affinity information to specific components of the structural complex (see Additional file 1: Text S1). We identified 955 ambiguous complexes, with an additional 20 complexes removed due to missing coordinates and/or steric clashes [37, 38]. Removing these complexes resulted in a filtered training database of 622 protein-protein dimers.

Consistent with results from previous studies [11, 19, 35, 39, 40], we found that removing complexes with ambiguous, missing or unreliable data was required to support robust training of affinity prediction models (see Additional file 1: Figure S1 and associated text). Models trained using either the complete PDBbind (1557 complexes) or the filtered database of 622 dimers performed very poorly when applied to the complete PDBbind dataset. For the remainder of this study, we therefore focus our analyses on the filtered PDBbind database of 622 dimers.

### Incorporating additional structural features improves protein-protein affinity prediction

**Fig. 1:**
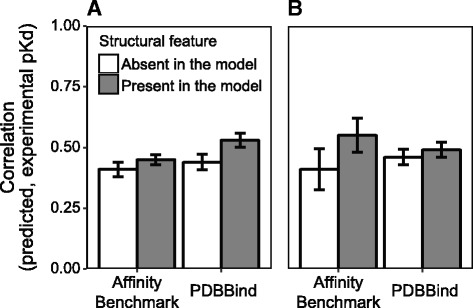
Including additional structural features improves prediction of protein-protein binding affinity. In addition to the atom-atom interaction terms evaluated in our previous study [22] we extracted additional features from protein-protein complexes in our filtered training datasets from PDBbind and the Binding Affinity Benchmark and performed cross-validation to evaluate the expected accuracy of affinity-prediction models trained using these features, when applied to new data (see Methods). We plot the Pearson correlation between predicted and experimentally-determined binding affinities for the original model (*white*) and the model including additional features (*gray*). Bars indicate standard errors. **a** Hydrophobicity and surface tension parameters were extracted from structural data and incorporated into the prediction model. **b** We calculated the root mean squared deviation (RMSD) between unbound and bound forms of the components of each protein-protein complex as well as differences in the area of each protein accessible to solvent (see Methods). These features were incorporated into prediction models. For complexes in the PDBbind database, we simulated the unbound forms by using homology modeling

We have previously developed statistical approaches for predicting protein-protein affinity incorporating a wide range of atom-atom interaction terms expected to impact macromolecular interactions [22]. However, in those analyses, protein-protein affinities could not be predicted with >0.49 correlation, after cross-validation. Applying these models to our filtered PDBbind dataset resulted in a correlation between predicted and experimentally-determined binding affinities of 0.44 in cross-validation analyses (Fig. 1a).

We did find that including additional atom-atom interaction terms can improve the accuracy of affinity-prediction models. For example, hydrophobicity [33] and surface tension parameters [34] are weakly correlated with binding affinity across the filtered PDBbind dataset (Spearman correlation >0.10, *p* < 7.48x10^−3^; Additional file 1: Figure S2A). Incorporating these parameters into the predictive model improved the correlation between predicted and experimentally-determined affinities in cross-validation analyses from 0.44 to 0.54 (one-tailed Fisher’s z = 2.04, *p* = 0.0207; Fig. 1a).

We also evaluated the relationship between binding affinity and structural changes caused by protein-protein binding by examining the change in conformational entropy upon complex formation. This can be roughly calculated by comparing the structure of the bound complex (*holo*) to the unbound (*apo*) structures and has been successfully applied for predicting binding affinity in a small dataset of 17 protein-protein complexes [41].

We extracted 143 *holo* complexes with corresponding *apo* structures from the protein-protein affinity benchmark database (Additional file 1: Table S2). Differences between *holo* and the *apo* forms were characterized by calculating root mean squared deviations (RMSDs) and changes in the accessible-to-solvent surface area upon complex formation. Although RMSD was not correlated with experimental binding affinity (Spearman correlation = 0.02, *p* = 0.73), we did observe a significant correlation between binding affinity and the change in accessible-to-solvent area caused by formation of the protein-protein binding interface, suggesting that this parameter may be useful for improving affinity prediction (Spearman correlation = −0.28, *p* = 8.63x10^−4^, Additional file 1: Figure S2B).

Cross-validation analysis confirmed that including changes in the accessible-to-solvent area as an explanatory variable improved affinity prediction accuracy, both on the Affinity Benchmark database (*r* ^2^ = 0.41 vs. 0.55; William’s test *p* = 2.3x10^−3^) and the filtered PDBBind database (*r* ^2^ = 0.46 vs. 0.49; William’s test *p* = 2.6x10^−3^; Fig. 1b).

Scoring functions that exhibit a Pearson correlation >0.72 and an RMSD <2 Å between predicted and experimental binding affinity in cross-validation analyses are commonly characterized as providing robust affinity inferences [11, 40, 42–44]. While our results do suggest that incorporating additional structural information can improve protein-protein affinity prediction, the improvements in accuracy we observed were generally incremental, and even best-case accuracy currently remains too low to support robust affinity inferences.

Existing studies of protein-protein affinity prediction have occasionally identified models capable of accurately predicting affinities on carefully curated small datasets, but accurate prediction of protein-protein affinity across large structure-affinity databases has remained unobtainable [23]. This could be due to lack of generalizability, possibly because curation of small datasets may inadvertently select for a subset of the structural features present in large-scale databases. Alternatively, the quality of both structural and affinity data in large databases could be highly variable, making some complexes more ‘difficult’ to accurately predict than others and potentially misleading model training procedures. Currently, almost nothing is known about how variation in characteristics of the structural and experimental data in structure-affinity databases might impact protein-protein affinity prediction. We examine this potential issue for the remainder of this study.

### Crystal resolution affects protein-protein affinity prediction accuracy

Crystallographic resolution is proportional to the precision of the 3-dimensional coordinates of the atoms in the structure. Typically, high-resolution structures (<2.5 Å) exhibit correct folding, have very small numbers of incorrect rotamers and present accurate surface loops. In contrast, low-resolution structures (>3.5 Å) are more likely to result in folding errors or incorrectly-modeled surface loops [45, 46]. We hypothesized that high-resolution structures would produce more reliable atom-atom interaction calculations and result in more accurate binding affinity predictions.

**Fig. 2:**
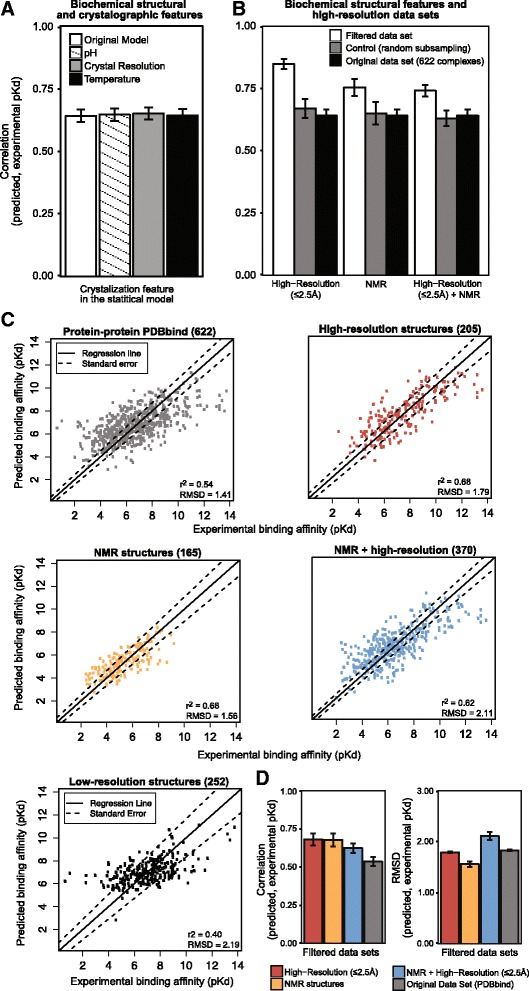
High-resolution structural information improves protein-protein binding affinity prediction. **a** We plot the Pearson correlation between predicted and experimentally-determined binding affinities (pKds) for the original affinity-prediction model incorporating only biochemical structural data (see Methods) and three models incorporating crystallographic features (temperature, pH and resolution) as additional parameters. See Methods for model training details. Bars indicate standard errors. **b** We trained affinity prediction models using high-resolution crystallographic data (≤2.5 Å), NMR structures or both high-resolution and NMR data. We plot the correlation between predicted and experimentally-determined affinities (pKds) for models trained using each type of filtered data set (*white series*) and compare results to models trained using the complete database of 622 protein-protein dimers (*black*) and models trained using randomly-selected subsets of the original data set of equal size to the high-resolution training data (*gray*). Bars indicate standard errors. **c** We performed leave-one-out cross-validation to evaluate the expected accuracy of affinity-prediction models applied to new data (see Methods). We plot the predicted vs. experimentally-determined binding affinities (pKds) of each cross-validated structural complex for models trained using the complete data set of 622 protein-protein dimers (*gray*), high-resolution crystallographic data (205 complexes with resolution ≤2.5 Å, red), 165 NMR complexes (*orange*) and the combined high-resolution + NMR data (370 complexes, *blue*). We report the best-fit regression line and its standard error as well as the Pearson correlation between predicted and experimentally-determined affinities (*r* ^2^) and the RMSD between predicted and experimental affinities. **d** plots the Pearson correlation and RMSD, respectively, for models trained using each type of filtered data set, with bars indicating standard errors

We did observe a weak but significant correlation between crystal resolution and the difference between predicted and experimental binding affinities (*r* ^2^ = 0.11, *p* = 5.26x10^−3^; Additional file 1: Figure S3A). However, including crystal resolution as a parameter in the affinity prediction model did not improve the correlation between predicted and experimental affinities, even across complexes included in the training dataset (*r* ^2^ = 0.64 vs. 0.65; Fisher one-tailed z = 0.16, *p* = 0.87; Fig. 2a). These results suggest that using crystal resolution as an explanatory variable in the model is unlikely to improve affinity prediction accuracy, even in the ‘best case’ scenario in which new data ‘look’ exactly like the data used for training.

However, constraining our training dataset to only include high-resolution structures (<2.5 Å, resulting in 205 protein-protein complexes) improved the correlation between predicted and experimental affinities on training data from 0.64 to 0.85 (Fisher one-tailed z = 6.14, *p* = 7.97x10^−10^; Fig. 2b). Furthermore, cross-validation analysis using only high-resolution structures resulted in increased predictive accuracy when applied to new data not included in training (*r* ^2^ = 0.54 vs. 0.68; Fisher two-tailed z = 2.85, *p* = 4.32x10^−3^; Fig. 2c,d).

Although Fisher’s z-transformation incorporates a correction for comparing results obtained on a subsample to results from the full dataset [47], we were concerned that selecting a subsample of the original testing data could lead to a spurious improvement in predictive accuracy, irrespective of the effects of higher crystal resolution. However, when we randomly selected subsets of equivalent size, accuracy never improved to the extent observed for the high-resolution dataset (*p* < 4x10^−3^; Fig. 2b).

Although these results suggest that training protein-protein affinity prediction using high-resolution structures may improve predictive accuracy, different types of complexes are likely to crystalize at different resolutions. If complexes whose affinities are more difficult to predict for inherent reasons also tend to crystalize at lower resolution, the effects of resolution on predictive accuracy may be indirect.

To address this issue, we grouped protein-protein complexes into clusters based on 90% sequence identity. Within each cluster of similar complexes, we calculated the correlation between crystal resolution and affinity prediction accuracy (Additional file 1: Table S3). Although there were only 21 clusters with >3 similar complexes in our dataset, we did find that all the clusters exhibiting a significant correlation between resolution and affinity prediction were consistent with the expectation that higher-resolution structures produced more accurate affinity predictions. While it is not possible to generalize from such limited data, these results do suggest that higher resolution structures may improve affinity prediction accuracy across at least some groups of similar protein-protein complexes.

Restricting training and testing data to either high-resolution or NMR data also resulted in a reduction of root-mean squared deviation (RMSD) between predicted and experimental affinities, compared to the original dataset (high-resolution and NMR RMSDs = 1.79 and 1.56, respectively, vs. 1.90 for the original dataset, *t*-test *p* < 0.03, Mann–Whitney *p* < 0.01; Fig. 2d). Together, these results suggest that training statistical models using high-resolution crystal structures or NMR data may improve affinity prediction accuracy when trained models are applied to new data.

It is interesting that restricting training data to NMR structures also improved predictive accuracy (see Fig. 2), as the resolution of NMR structures is typically lower than X-ray crystal structures. However, NMR structures are determined from proteins in solution, which may more accurately reflect the native functional environment of the protein [48, 49]. The capacity to capture protein-protein interactions in solution may contribute to the improved predictive accuracy of models trained using NMR data, particularly for cases in which the crystallographic process might introduce structural artifacts.

Experimental conditions such as temperature and pH are critical for the formation and stability of a protein crystal [50]. However, the optimal conditions for crystallization may differ from those used for measuring binding affinity, potentially creating a mismatch between a crystalized protein-protein complex and that same complex in experimental solution. To examine the potential effects of crystallization conditions on protein-protein affinity prediction, we extracted temperature and pH information from the Protein Data Bank [51] for the complexes in our training dataset and evaluated the effect of including this information on predictive accuracy. Although both crystallization temperature and pH were weakly negatively correlated with experimental binding affinity (*r* ^2^ = −0.26, *p* = 4.91x10^−10^ and *r* ^2^ = −0.16, *p* = 1.96x10^−4^ for temperature and pH, respectively. Additional file 1: Figure S3B), we observed no improvement in predictive accuracy when these parameters were incorporated into the statistical model (Fisher one-tailed z < 0.1, *p* > 0.92; Fig. 2a).

Overall, these results suggest that the quality of structural data can affect the accuracy of statistical affinity prediction, and that training models using high-quality structures may be one avenue available to improve protein-protein and other affinity predictions. While limiting training data to high-resolution structures was not required for accurate prediction of protein-small molecule or protein-DNA/RNA affinities in our previous analysis [22], protein-protein complexes typically have larger numbers of atoms at the protein-ligand interface and may be more sensitive to potential errors induced by lower crystal resolution. Differences in crystal resolution across protein-small molecule, protein-DNA/RNA and protein-protein training datasets may also contribute to differences in affinity prediction accuracy.

### Lack of information on binding assay conditions impairs protein-protein affinity prediction

In addition to crystallographic conditions or resolution, variation in the experimental conditions and assays used to measure binding affinity could affect prediction accuracy. Different proteins can have dramatically different activities across temperature, pH and concentration of ions or cofactors [52, 53], and assay conditions have been shown to strongly affect reaction rates [54–56].

Even though experimental conditions can be critical for evaluating affinity measurements, this information is not available in the major structure-affinity databases used for training statistical predictors [30, 57]. Detailed experimental information is available for a small protein-protein affinity benchmark database [28]. After excluding complexes with missing data, we obtained 127 protein-protein complexes with data indicating the pH of the binding affinity experiment and 103 complexes with temperature information (Additional file 1: Table S2).

**Fig. 3:**
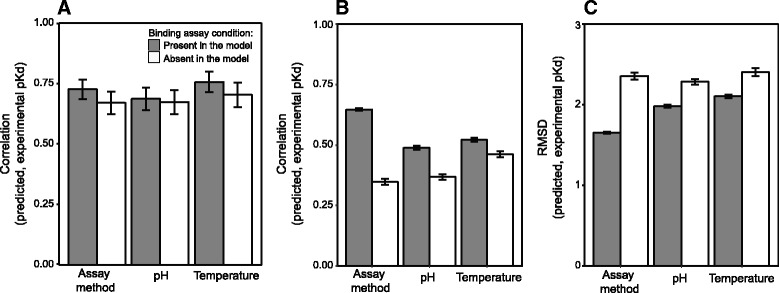
Incorporating information about binding assay conditions improves protein-protein affinity prediction. **a** We plot the Pearson correlation between predicted and experimentally-determined binding affinities (pKd) using models with (*white*) and without (*gray*) three features describing the conditions under which binding affinities were measured experimentally (temperature, pH and the assay method). **b** We plot the correlation between predicted and experimentally-determined binding affinities for the same models examined in (**a**), using unseen testing data generated by leave-one-out cross validation (see Methods). **c** We plot the RMSD between predicted and experimentally-determined binding affinities for the same models in (**a** and **b**), using leave-one-out cross-validation. In each panel, bars indicate standard errors

We found no significant increase in the correlation between predicted and experimental affinities when binding experiment pH was included as an explanatory variable, even across data used to train the model (*r* ^2^ = 0.67 vs. 0.69, William’s t = 0.43, *p* = 0.34; Fig. 3a). Although pH has been shown to affect binding affinity measurements in some systems [58–60], the effect of pH on pKd may depend on particular properties of the specific interacting proteins. We did observe a small, marginally-significant increase in correlation when including temperature in the model (*r* ^2^ = 0.70 vs. 0.76, William’s t = −1.81, *p* = 0.04; Fig. 3a). Cross-validation analysis confirmed that binding assay temperature has the capacity to improve affinity prediction accuracy when applied to unseen testing data (*r* ^2^ = 0.46 vs. 0.52; William’s t = −29.84, *p* = 9.03x10^−188^; Fig. 3b).

Although the dataset examined in this analysis was small, compared to the training data available in the filtered PDBbind data set (~100 vs. ~600 complexes), these results suggest that incorporating parameters describing the experimental conditions used to measure protein-protein binding affinity may be important for training affinity-prediction models. The lack of information describing binding assay conditions in large structure-affinity databases may impose a limit on the accuracy of statistical models constructed from these databases.

Protein-protein affinity can be measured by a variety of approaches, some of which may more strongly impact affinity prediction than others. Common technologies used in the protein-protein affinity benchmark database [28] are Isothermal Titration Calorimetry (ITC [61]), surface plasmon resonance (SPR [62]), and inhibition assays [63–66].

We observed a weak but significant correlation between the use of inhibition assays and experimentally-determined affinity values (Spearman correlation = 0.33, *p* = 1.16x10^−4^), whereas ITC was weakly negatively correlated with affinity (Spearman correlation = −0.36, *p* = 1.84x10^−5^; Additional file 1: Figure S3C). These results suggest that reported affinity measurements are somewhat dependent on the type of assay used: inhibition assays typically result in higher affinities, whereas ITC tends to produce lower affinity values. It is not clear whether this “assay effect” represents a general bias in one or more of the methodologies used to assess binding affinity, or if different methodologies tend to be applied to complexes with higher vs. lower biological affinities.

When we included experimental assay method as an explanatory variable in the statistical model, we observed a strong increase in predictive accuracy, assessed by cross-validation (*r* ^2^ = 0.35 vs. 0.65; William’s t = −29.84, *p* = 9.03x10^−188^; Fig. 3b). In addition, there was no significant difference between training and cross-validation correlation results (Fisher’s z = 1.22, *p* = 0.11; Fig. 3a,b), suggesting that the optimized statistical model—including assay method—exhibits minimum over-fitting.

Incorporating binding assay conditions in our statistical model also resulted in a significant decrease in RMSD between predicted and experimentally-determined affinities (Fig. 3c). Adding binding assay pH to the statistical model reduced RMSD from 2.28 to 1.98 (*t*-test t = −7.61, *p* = 2.09x10^−12^; Mann–Whitney w = 2272, *p* = 2.66x10^−11^). Similarly, RMSD decreased from 2.40 to 2.10 when temperature was incorporated (*t*-test t = −5.80, *p* = 4.41x10^−8^; Mann–Whitney w = 3221, *p* = 1.39x10^−5^). Finally, RMSD decreased from 2.36 to 1.65 when binding assay method was included as a model parameter (*t*-test t = −15.58, *p* = 2.80x10^−30^; Mann–Whitney w = 383, *p* = 1.65x10^−29^).

Overall, these results suggest that incorporating information about the experimental conditions used to measure protein-protein affinity can have a strong effect on the predictive accuracy of statistical models. Detailed experimental conditions are generally not incorporated into large-scale structure-affinity databases, which may place a practical upper bound on the accuracy of statistical affinity prediction.

### Could database errors limit predictive accuracy?

Manual examination of specific examples in the protein-protein affinity benchmark database [28] revealed that, in some cases, the conditions of the crystalized complex are so different from the conditions of the binding assay that it is not clear they are biochemically comparable. For example, the crystal structure of Nuclease A (NucA) in complex with intracellular inhibitor NuiA is a D121A mutant (PDB ID 2O3B), whereas the affinity assay was performed using the wild-type NuiA [67]. This particular complex was the 3^rd^ worst prediction made by our statistical model, with a predicted pKd based on the mutant structure of 7.06 vs. an experimental pKd of the wild-type protein of 11.49. It is not clear whether this mis-prediction is due to a poor statistical fit to this complex or to differences between the binding affinities of the mutant vs. wild-type proteins.

**Fig. 4:**
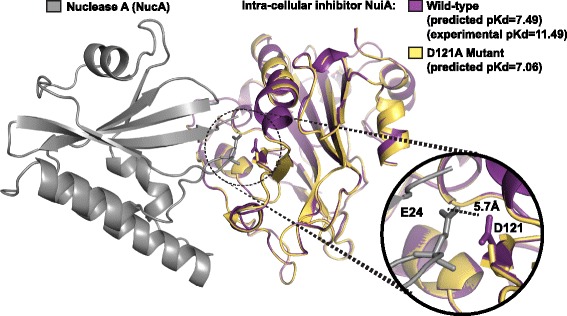
Database errors may interfere with affinity prediction accuracy. We predicted the binding affinities of mutant D121A intracellular inhibitor NuiA (NuiA; *yellow*) and wild-type NuiA (*purple*) in complex with Nuclease A (nucA; *gray*) using the trained statistical affinity-prediction model. The wild-type NuiA structure was inferred by homology modeling, using the mutant structure as a template. We plot the structure of each complex and report predicted and experimentally-determined binding affinities (pKds). Inset displays a close-up of the D121A mutation, showing an inferred hydrogen bond between D121 and E24 of NucA (*dashed line*)

To evaluate the potential effects of this mismatch on predicted binding affinity accuracy, we generated the wild-type structure of NuiA using homology modeling [68] and re-estimated the binding affinity using the trained statistical model. Modeling the wild-type NuiA in complex with NucA increased the predicted pKd from 7.06 (mutant NuiA) to 7.49, decreasing the difference between predicted and experimentally-determined affinity somewhat, possibly because the D121A mutation disrupted a hydrogen bond between wild-type NuiA’s D121 and NucA’s E24 (Fig. 4).

Although the significance of the improvement in this single ‘case study’ cannot be evaluated statistically—and is likely to depend on the specific scientific question being considered—this result does suggest that small differences between the crystalized protein-protein complex and the complex whose binding affinity is measured—in this case a single amino acid mutation—may have a measurably negative affect on the accuracy of affinity prediction. The extent of similar errors in large-scale structure-affinity databases is unknown.

Missing information about key affinity-determining factors from either the crystallization or affinity experiments could also affect affinity prediction accuracy. In one example, the crystal structure of GTP-Bound Rab4Q67L GTPase in complex with the central Rab binding domain of Rabenosyn-5 (PDB ID 1Z0K) has an additional cofactor, 2-(N-morpholino)-ethanesulfonic acid or MES, which was not present in the binding assay [69]. This complex was the 4^th^ worst prediction made by the statistical model (predicted pKd = 9.14; experimental pKd = 5.11). Although the extent to which the presence/absence of the MES cofactor may have affected affinity prediction is unclear, that the crystalized complex does not correspond to the complex assayed in the experimental affinity measurement raises concerns about the accuracy of this database entry.

Other examples of missing information likely to affect affinity prediction could be manually identified from the affinity benchmark database [28], many of which appear to have had a negative impact on affinity predictions made by our statistical model (see Additional file 1: Text S2). Overall, we found 4 of the 10 worst predictions made by the statistical model were for complexes with obvious mismatches between crystallization and binding assay conditions or cases in which information potentially impacting affinity measurement or crystallization was missing from the database (Additional file 1: Table S4).

These potential database errors were identifiable due to the amount of detail provided in small curated databases like the protein-protein affinity benchmark [28]. However, we expect similar potential issues exist in large-scale databases like PDBbind and BindingDB, which together contain >10,000 protein-ligand structures [30, 57]. Many of the entries in these large structure-affinity databases lack information concerning binding assay and/or crystallization conditions. Our results suggest that this information may be critical for supporting accurate, high-throughput affinity prediction and also important for identifying potential database errors.

We also identified a handful of cases in which binding affinity values were incorrectly entered in the PDBbind database. For example, the binding affinity (pKd) assigned to Human prolactin (hPRL) in complex with its receptor is 0.67 in PDBbind, whereas the experimentally-determined binding affinity from the literature is >5.65, depending on pH [70]. Similarly, the prolactin and prolactin receptor mutant complex has an assigned pKd of 1.03 in PDBbind, whereas the affinity from the literature is 6.14 [70]. Although these particular cases were manually corrected in the filtered dataset used for this study, the extent to which various errors are present in large structure-affinity databases remains unknown, making it difficult to characterize the potential effects of database errors on affinity prediction.

## Discussion

The accuracy of machine learning and other statistical prediction methods depends on having a large quantity of high-quality training data. Errors in the training data can impair the inferred model’s predictive performance [71], whereas a too-small training dataset can interfere with generalizability to new data [72]. Our results suggest that curation errors, lack of information about experimental conditions and low-quality data present in large structure-affinity databases could reduce the maximum achievable accuracy of protein-protein affinity prediction models developed from these databases.

We have shown that limiting training data to high-resolution crystal structures—easily extracted from structural information—can dramatically improve affinity prediction. However, we are cautious that the resulting reduction in breadth of training data may limit the generalizability of inferred models to new problems, particularly complex structural interactions that may not crystalize at high resolution due to inherent flexibility.

We have also shown that incorporating information about the experimental conditions used to measure binding affinity may be important for producing accurate affinity predictions from structural data, probably due to their effects on resulting affinity measurements. Unfortunately, most large structure-affinity databases do not include detailed experimental information, and databases that do include this information appear to have at least some examples of dramatic mismatches between crystallographic and affinity-measurement conditions. The extent to which these types of potential errors are present in large-scale databases is not known, making it difficult to assess the general impact of these potential problems on affinity prediction.

## Conclusion

Although careful manual curation can be used to develop high-quality structure-affinity databases, this approach is unlikely to scale up to the number of structures required for training robust, generalizable predictive models. A possible computational approach to building high-quality, large-scale structure-affinity databases would be to extract detailed information about crystallographic and affinity-measurement conditions directly from scientific literature using text-mining approaches [73–75], although errors in text-mining could then potentially propagate to training databases. Alternatively, authors could be encouraged to directly supply the required information as part of a database submission policy associated with scientific publication. This approach has been successfully used to develop the Protein Data Bank [32], Genbank [76] and similar community resources. Ultimately, it may be up to the community of researchers to develop the standards and practices necessary to support large-scale investigations of the general structural basis for protein-protein interactions.

## Additional file

Additional file 1: Appendix with Supplementary Material. Appendix, including text, tables, and figures for supplementary data. (PDF 1564 kb)
