# Supplementary material for: Improving the accuracy of high-throughput protein-protein affinity prediction may require better training data
Source: BMC Bioinformatics. 2017 Mar 23;18(Suppl 5):102. doi: 10.1186/s12859-017-1533-z (PMC5374557; doi:10.1186/s12859-017-1533-z)
Supplement: Supplementary file 1 — Appendix with Supplementary Material. Appendix, including text, tables, and figures for supplementary data. (PDF 1564 kb) [file 12859_2017_1533_MOESM1_ESM.pdf]

Supplementary Materials

Raquel Dias and Bryan Kolaczkowski

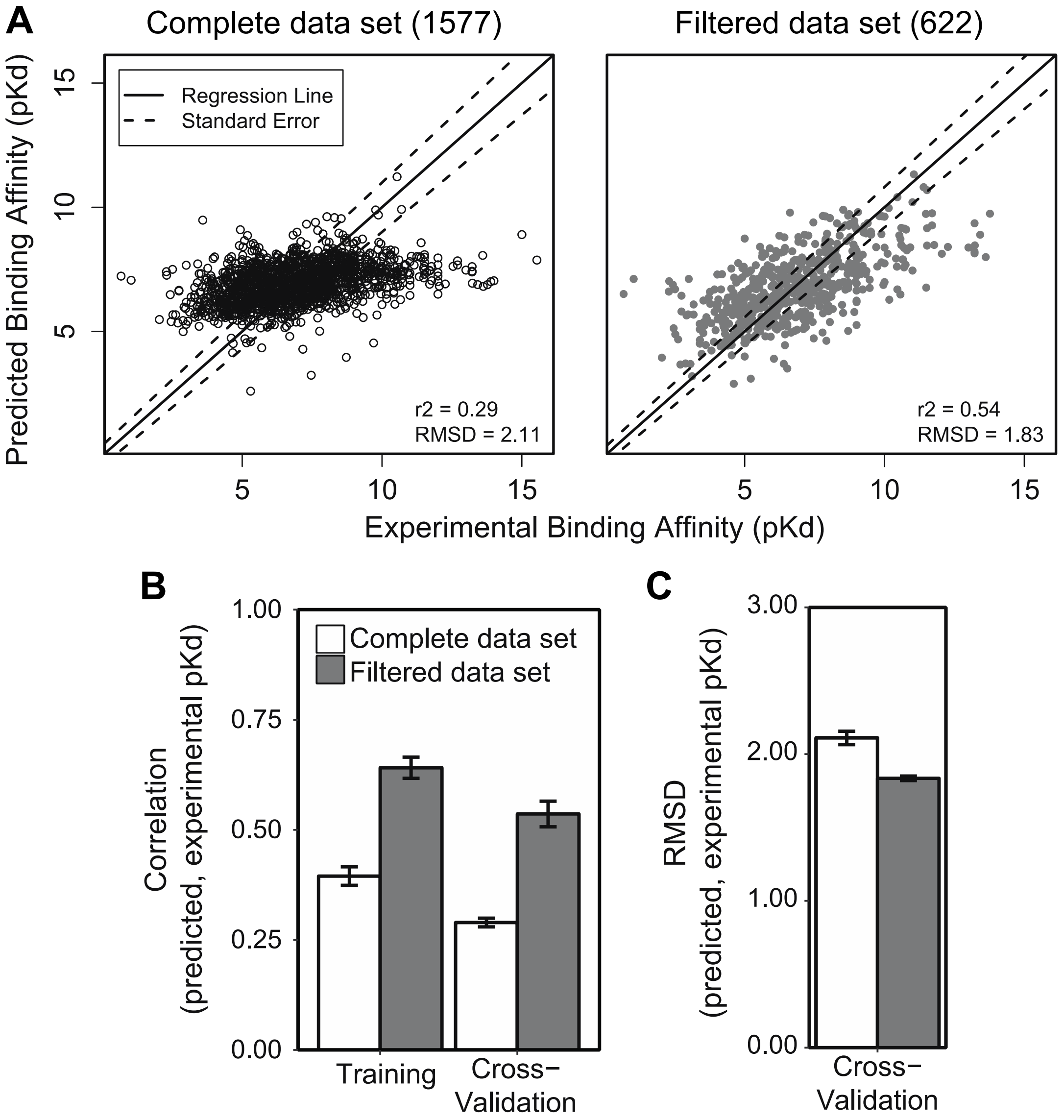

Figure S1. Filtering ambiguous cases from PDBbind improved prediction of protein-protein binding affinity. We trained statistical models and predicted binding affinities using the complete PDBbind database (1577 complexes) and filtered dataset in which database entries with multimeric complexes or ambiguous affinity information were excluded (622 complexes). We measured the correlation and root mean squared deviation (RMSD) between predicted and experimental affinities obtained using either dataset. In order to estimate the binding affinities of

the ambiguous cases of structures with multiple chain pairs, we selected the chain pairs with the largest number of intermolecular interactions. **A.** We plot the correlation between predicted and experimentally-determined binding affinities using the unfiltered (left) and filtered (right) PDBbind data to train predictive models. Correlations and RMSDs are reported on unseen testing data after cross-validation (see Methods). We plot the best-fit regression line and its standard error (dotted lines). **B.** We plot the correlation between predicted and experimental binding affinities using the complete PDBbind (white) and the filtered dataset (gray). Series show correlations across training data and on unseen testing data after cross-validation. Bars indicate standard errors. **C.** We plot the RMSD between predicted and experimental binding affinities for models trained using the complete PDBbind (white) And the filtered dataset (gray) after cross-validation. Bars represent RMSD standard errors.

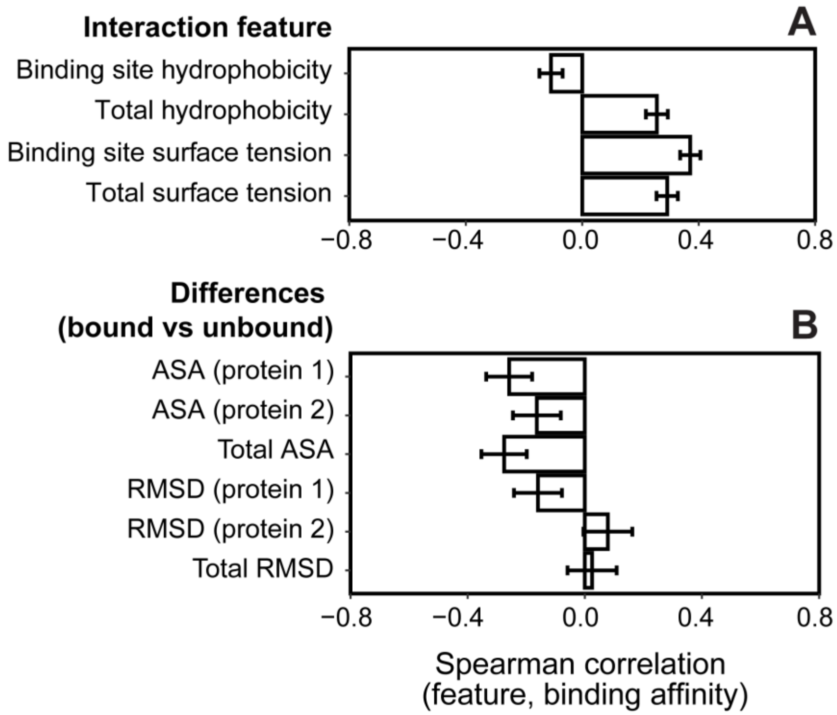

Figure S2. Structural features and differences between bound and unbound complexes are correlated with experimental binding affinity. We plot the Spearman correlation between the experimental binding affinity and a set of features extracted from crystal structure information and binding assay conditions. Error bars represent correlation standard errors. **A.** Spearman correlation between experimental binding affinity and additional intermolecular interaction terms included in the statistical prediction model (see Methods for feature details). **B.** Spearman correlation between experimental binding affinity and differences between bound and unbound protein structures (see Methods for details about structural calculations). RMSD=root mean squared deviation; ASA=accessible to solvent area. Protein 1 is taken as the smaller of the two proteins in the binary complex.

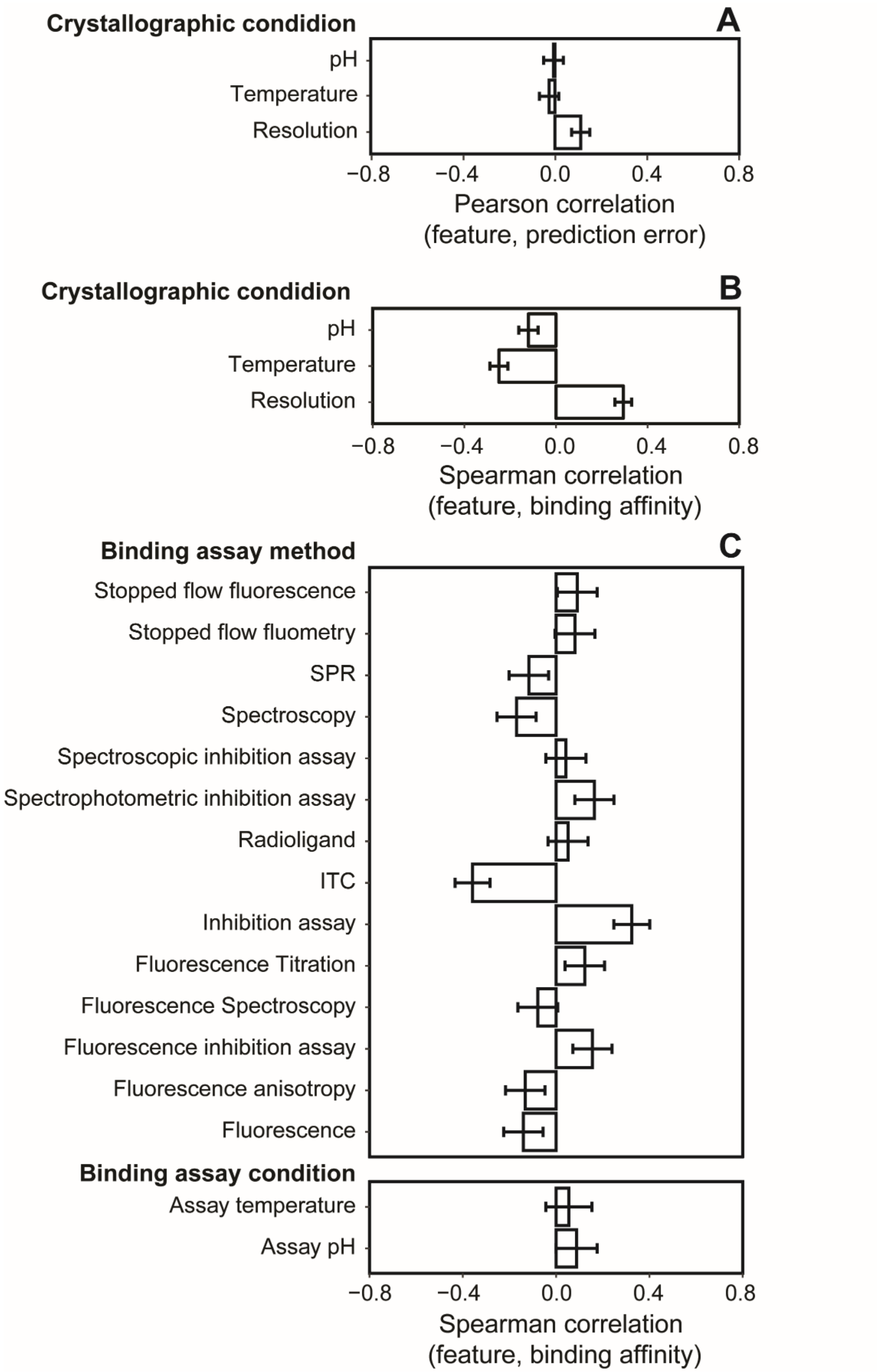

Figure S3. Crystallographic conditions and binding assay method are correlated with experimental binding affinity. We plot the correlation between experimental binding affinity and a set of features extracted from crystal structure information and binding assay conditions.

Bars indicate correlation standard errors. **A.** We plot the Pearson correlation between prediction error and crystallographic conditions (Temperature, pH and crystal resolution). Prediction error represents the difference between predicted and experimental binding affinities. **B.** We plot the Spearman correlation between experimental binding affinity and crystallographic conditions (Temperature, pH and crystal resolution). **C.** We plot the Spearman correlation between experimental binding affinity and various binding assay conditions, including Temperature, pH and the method used to assess binding affinity (see Methods for details).

Text S1. Additional details on the effects of filtering ambiguous complexes from the PDBBind database.

In order to evaluate the effects of filtering ambiguous complexes, we trained a statistical model using either the complete PDBbind or our curated database of 622 protein-protein dimers and used it to predict binding affinities across the complete PDBbind database (1577 complexes). Because ambiguous complexes consist of multiple chains with no information about which pairs of chains correspond to binding affinity measurements, we selected the chain pair with the highest number of favorable intermolecular interactions for each prediction.

We evaluated each model by measuring the correlation (r^2) and RMSD between predicted and experimental binding affinities obtained across both the complete PDBbind and the filtered dataset of 622 protein-protein dimers, using 100 replicates of leave-one-out cross-validation to assess expected predictive accuracy and possible model over-fitting (see Methods).

As expected, the correlation between experimental and predicted binding affinities was significantly lower for the unfiltered data set, compared to the filtered data set (r^2=0.29 vs. 0.54; Fisher's z=6.33, p=2.41×10^{-10}; Supplementary Fig. S1A,B). The RMSD between experimental and predicted affinities was also significantly larger for the unfiltered data set, compared to the filtered data set (RMSD=2.11 vs. 1.83; Mann-Whitney w=7406, p=4.16×10^{-09}; Supplementary Fig. S1A,C). Together, these results suggest that ambiguity in structure-affinity training databases can negatively impact protein-protein affinity prediction accuracy.

Text S2. Additional details on the errors in the binding affinity databases that may disrupt the prediction of protein-protein binding affinity.

The affinity of human H-Ras for GTPase-activating protein p120GAP (GAP-334) has been shown to strongly depend on the concentration of specific ions in solution [1], but the concentration of these ions is not available from the crystal structure (PDB ID 1WQ1). This complex was the 7th worst prediction made by the statistical model, with a difference of 3.85 between predicted and experimental binding affinity (pKd=8.82 vs. 4.77, respectively). Although it is difficult to reliably evaluate the potential effects of differences in ion concentrations or types on prediction accuracy, the lack of information about an important variable known to affect affinity measurement raises potential concerns.

The crystal structure of colicin E9 DNAse domain with its cognate immunity protein IM9 contains phosphate as a cofactor (PDB ID 1EMV), but there is no information on the presence/absence

of this cofactor in the corresponding binding assay. In addition, the binding assay for this protein-protein complex had 200mM NaCl [2], whereas the crystal structure has no information on salt concentration. This complex had the largest difference between predicted and experimental affinities in our analysis (pKd=13.62 vs. 8.70, respectively).

Other examples of mismatches between crystallization and affinity-measurement conditions include the actin-depolymerizing factor homology domain in complex with actin, which was crystallized with adenosine diphosphate (PDB ID 3DAW), whereas affinity was measured in the presence of adenosine triphosphate [3]. The SAP/FynSH3/SLAM ternary complex shows Fyn interacting with SAP and SLAM (PDB ID 1M27), whereas affinity was measured for the Fyn/SAP binary complex [4]. In another example, the crystal structure of the heterodimeric complex of XRCC1 and DNA ligase III-alpha BRCT domains consists of mouse XRCC1 and human ligase (PDB ID 3PC8), whereas the binding assay was performed for human XRCC1 and human ligase [5].

Table S1. Protein-protein data set. After removing complexes with ambiguous binding-affinity measurements or multiple ligands, our filtered database contained 622 protein-protein dimers.

PDB ID	Resolution	Affinity data	-log (Kd, Ki, IC50)	Release year	Protein name
1a22	2.6	Kd = 0.34nM	9.47	1998	GROWTH HORMONE RECEPTOR
1abt	NMR	Kd = 1.4uM	5.853872	1994	a-bungarotoxin (BGTX)
1acb	2	Kd = 0.2nM	9.7	1993	bovine alpha-chymotrypsin
1an1	2.03	Ki = 0.9nM	9.05	1998	TRYPSIN
1atn	2.8	Kd = 0.45nM	9.35	1992	rabbit skeletal muscle G-actin
1avp	2.6	Kd = 12nM	7.92	1997	adenoviral proteinase
1avx	1.9	Kd = 60fM	13.22	1998	Porcine trypsin
1axi	2.1	Kd = 14nM	7.85	1998	GROWTH HORMONE RECEPTOR(mutant)

1ay7	1.7	Kd = 1uM	6	1999	RIBONUCLEASE SA
1aze	NMR	Kd = 60uM	4.22	1999	growth factor receptor-bound protein 2 (grb2)
1azg	NMR	Kd = 16uM	4.79588	1998	Fny(SH3 domain, 82 - 148)
1bjr	2.44	Ki = 21nM	7.68	1998	proteinase k
1buh	2.6	Kd = 77nM	7.11	1998	HUMAN CDK2 KINASE
1bvn	2.5	Ki = 9pM	11.05	1998	PIG PANCREATIC ALPHA-AMYLASE
1bxl	NMR	Kd = 0.34uM	6.468521	1997	BCL-XL
1c9p	2.8	Ki = 1.0nM	9	2000	PORCINE TRYPSIN
1cka	1.5	Kd = 1.9uM	5.72	1995	c-crksh3-n
1ckb	1.9	Kd = 5.2uM	5.28	1995	c-crksh3-n
1clv	2	Kd = 1nM	9	2000	TMA
1cwb	2.2	Ki = 13nM	7.886056	1996	cyclosporin A (CsA)
1cwc	1.86	IC50 = 16.0nM	7.8	1996	cyclophilin a
1cyn	1.85	Ki < 21nM	7.68	1996	cyclophilin b
1czq	1.5	IC50 = 29uM	4.537602	1999	IQN17
1d4t	1.1	Kd = 650nM	6.187087	1999	SAP
1d6r	2.3	Kd = 0.13nM	9.89	2000	bovine TRYPSIN
1ddm	NMR	Kd =	5.77	2000	numb protein

		1.7uM			
1dfj	2.5	Ki = 59fM	13.23	1997	Ribonuclease A
1dhk	1.85	Ki = 35pM	10.46	1997	PORCINE PANCREATIC ALPHA-AMYLASE
1djs	2.4	Kd = 41nM	7.39	2000	FIBROBLAST GROWTH FACTOR RECEPTOR 2
1dp5	2.2	Ki = 0.9nM	9.05	2000	PROTEINASE A
1dpj	1.8	Ki = 3nM	8.522879	2000	Aspartic proteinase A from yeast
1dpu	NMR	Kd = 1uM	6	2000	RPA32(C-terminal domain of Replication protein A,172-270)
1E96	2.4	Kd = 2.7uM	5.57	2000	ras-related c3 botulinum toxin substrate 1
1ees	NMR	Kd = 20nM	7.7	2000	GTP-BINDING PROTEIN Cdc42Hs
1ej4	2.25	Kd = 50nM	7.3	2000	eukaryotic initiation factor 4e
1eja	2.7	Ki = 1.0nM	9	2001	PORCINE beta -TRYPSIN
1emv	1.7	Kd = 24fM	13.62	2000	endonuclease domain of colicin E9
1eoj	2.1	Ki = 17fM	13.77	2000	thrombin alpha
1eol	2.1	Ki = 38fM	13.42	2000	thrombin alpha
1es0	2.6	IC50 = 0.08uM	7.1	2000	GAD(207-220)
1lcj	1.8	Kd = 1nM	9	1995	P56 LCK Tyrosine kinase
1ldt	1.9	Ki = 1.8nM	8.74	1998	PORCINE TRYPSIN
1om2	NMR	Kd = 20uM	4.69897	2000	rat Tom20(translocases of outer mitochondrial membranes)

1sfi	1.65	Ki = 0.1nM	10	1999	bovine ¬¶√Ç-trypsin
1shc	NMR	Kd = 53nM	7.28	1997	shc
1vrk	1.9	Kd = 6.8nM	8.167491	1999	E84K-CALMODULIN
1f34	2.45	Kd = 0.1nM	10	2001	porcine pepsin A
1f3v	2	Kd = 7.8uM	5.11	2000	TRADD-N
1f47	1.95	Kd = 21.6uM	4.665546	2001	cell division protein ZipA/M185
1f5r	1.65	Ki = 65uM	4.19	2001	rat Delta I16V17/Q156K trypsinogen
1f7z	1.55	Ki = 12.4uM	4.91	2001	rat K15A trypsinogen
1fc2	2.8	Kd = 22.5nM	7.65	1981	Fragment B of Protein A from Staphylococcus Aureus
1ff1	NMR	Kd = 12uM	4.920819	2000	epidermal growth factor receptor substrate 15(EH2 of Eps15)
1fle	1.9	Ki = 6nM	8.22	1997	PORCINE PANCREATIC ELASTASE
1fmo	2.2	Ki = 2.3nM	8.64	1998	camp-dependent protein kinase
1fy8	1.7	Ki = 9uM	5.05	2000	rat Delta I16V17 trypsinogen
1g0v	2	Ki = 0.1nM	10	2001	yeast PROTEINASE A
1g1e	NMR	Kd = 29nM	7.537602	2000	mammalian Sin3A PAH2 domain
1g5j	NMR	Kd = 0.6nM	9.221848	2001	the anti-apoptotic protein Bcl-xL
1g6v	3.5	Kd = 72nM	7.14	2000	bovine carbonic anhydrase
1g9i	2.2	Kd =	6.920819	2000	bovine beta-trypsin

		0.12uM			
1gag	2.7	Ki = 750nM	6.12	2001	insulin receptor, tyrosine kinase domain
1gbq	NMR	Kd = 3.5mM	2.46	1997	growth factor receptor-bound protein 2 (grb2)
1gl0	3	Ki = 0.8nM	9.1	2001	BOVINE ALPHA-CHYMOTRYPSIN
1gla	2.6	Kd = 0.18uM	6.74	1993	Glycerol kinase
1grn	2.1	Kd = 388nM	6.41	1999	CDC42 GTPase
1gua	2	Kd = 40nM	7.4	1997	rap1a
1guw	NMR	Kd = 1.9uM	5.721246	2002	heterochromatin protein 1 (HP1)
1h0t	NMR	Kd = 6uM	5.22	2003	IMMUNOGLOBULIN G BINDING PROTEIN A
1h1v	3	Kd = 23nM	7.64	2003	Actin
1h3h	NMR	Kd = 220nM	6.657578	2003	mouse Gads T cell adaptor C-terminal SH3 domain
1h59	2.1	Kd = 37nM	7.43	2002	IGFBP-5
1h6e	3.6	Kd = 0.7uM	6.154902	2001	AP50
1haa	NMR	IC50 = 2nM	8.69897	2001	¬¶√Å-bungarotoxin(¬¶√Å-BTX) from Bungarus multicinctus venom (Sigma)
1he8	3	Kd = 2.5uM	5.6	2001	Ras GTPase G12V mutant
1i3z	2.15	Kd = 131nM	6.882729	2003	EWS/FLI1 activated transcript 2(ETA-2)
1i4e	3	Kd = 0.12uM	6.920819	2001	CASPASE-8
1i5h	NMR	Kd = 53uM	4.275724	2001	Ubiquitin ligase Nedd4(WW¬¢√≥ domain)

1i8h	NMR	Kd = 29uM	4.537602	2001	Pin1 WWdomain
1idg	NMR	Kd = 65nM	7.187087	2001	¬¶√Å-Bungarotoxin(Bgtx)
1irs	NMR	Kd = 6uM	5.221849	1997	PTB domain of insulin receptor substrate-1(IRS-1)
1iwq	2	Kd = 8.8nM	8.055517	2003	Ca2+-Calmodulin
1j19	2.4	Kd = 16.4nM	7.785156	2003	mouse radixin FERM domain
1j2j	1.6	Kd = 1.4uM	5.85	2003	adp-ribosylation factor 1
1j4p	NMR	Kd = 10.2uM	4.99	2001	protein kinase spk1
1j4q	NMR	Kd = 0.36uM	6.44	2001	protein kinase spk1
1j7d	1.85	Kd = 2uM	5.69897	2001	UBIQUITIN-CONJUGATING ENZYME E2-17 KDA, hUbc13
1j7v	2.9	Kd = 35pM	10.46	2001	IL-10R1(N29,53,89,133,156,168Q)
1j7z	2.25	Kd = 0.02uM	7.69897	2001	Ribonuclease S(RNase S)
1j80	2.1	Kd = 0.3uM	6.522879	2001	Ribonuclease S(RNase S)
1j81	2.2	Kd = 0.08uM	7.09691	2001	Ribonuclease S(RNase S)
1jbd	NMR	IC50 = 0.12uM	6.920819	2001	Alpha-bungarotoxin
1jgn	NMR	Kd = 0.35uM	6.455932	2003	C-terminal domain of poly(A)-binding protein (PABC)
1jh4	NMR	Kd = 1.5uM	5.823909	2003	C-terminal domain of poly(A)-binding protein (PABC)
1jiw	1.74	Kd = 4pM	11.4	2001	alkaline protease
1jm4	NMR	Kd =	5	2002	p300/CBP-associated factor (PCAF)

		10uM			
1jmq	NMR	Kd = 40uM	4.4	2001	65 kda yes-associated protein
1jsp	NMR	Kd = 50uM	4.30103	2002	tumor protein p53 peptide
1jtd	2.3	Ki = 27.2pM	10.57	2001	TEM-1 beta-lactamase
1k3n	NMR	Kd = 10.2uM	4.9914	2001	FHA1 Domain of Rad53
1k3q	NMR	Kd = 0.3uM	6.522879	2001	FHA1 Domain of Rad53
1ka7	NMR	Kd = 0.60uM	6.22	2001	sh2 domain protein 1a
1kac	2.6	Kd = 14.8nM	7.83	1999	Adenovirus fiber knob protein
1kbh	NMR	Kd = 34nM	7.468521	2002	CREB-BINDING PROTEIN
1kna	2.1	Kd = 7uM	5.15	2002	heterochromatin protein 1
1kne	2.4	Kd = 2.5uM	5.6	2002	heterochromatin protein 1
1ktz	2.15	Kd = 290nM	6.54	2002	Human TGF-beta Type II Receptor Extracellular Domain
1kxp	2.1	Kd = 1nM	9	2002	actin, alpha skeletal muscle
1l0a	2.9	Kd = 23.9uM	4.621602	2002	human tumor necrosis factor receptor(TRAF3)
1l2z	NMR	Kd = 203uM	3.69	2002	cd2 antigen (cytoplasmic tail)-binding protein
1l4d	2.3	Kd = 196.6nM	6.71	2002	SKa delta
1l4z	2.8	Kd = 196.6nM	6.71	2002	SKa delta (Q5E,W6A)
1l8c	NMR	Kd = 7nM	8.154902	2002	CREB-binding protein TAZ1 domain

1lp1	2.3	Kd = 2uM	5.7	2003	protein Z(A1V,G29A)
1lw6	1.5	Kd = 2pM	11.7	2002	subtilisin BPN
1lx5	3.3	Kd = 1.2nM	8.92	2003	ActRII
1lxh	NMR	Kd = 65nM	7.187087	2002	Cbtx
1lzw	2.5	Kd = 0.33uM	6.481486	2002	adapter protein ClpS
1m10	3.1	Kd = 5.8nM	8.24	2002	Glycoprotein Ib- alpha (N21,159Q,M239V)
1m1e	2.1	Kd = 1nM	9	2002	beta-catenin
1m5n	2.9	Kd = 2.1nM	8.677781	2003	importin beta
1mah	3.2	Kd = 25pM	10.6	1996	acetylcholinesterase (mAChE)
1mcv	1.8	Ki = 98nM	7.008774	2003	porcine pancreatic elastase (PPE)
1mik	1.76	IC50 = 450nM	6.35	1996	cyclophilin a
1mv0	NMR	Kd = 0.0933mM	4.030118	2003	Bin1C SH3 domain
1mxl	NMR	Kd = 154uM	3.812479	1999	human Cardiac Troponin-C(cNTnC¬°¬§Ca2+)
1mzw	2	Kd = 1.97uM	5.71	2003	CypH
1nlo	NMR	Kd = 3.4uM	5.47	1997	c-src tyrosine kinase
1nlp	NMR	Kd = 11uM	4.96	1997	c-src tyrosine kinase
1ntv	1.5	Kd = 1.5uM	5.82	2003	disabled homolog 1

1nw9	2.4	Ki = 0.013uM	7.89	2003	Caspase-9
1o9a	NMR	Kd = 1uM	6	2003	streptococcal (S. dysgalactiae) FnBP peptide (B3)6,7
1oc0	2.28	Kd = 1nM	9	2003	Plasminogen Activator Inhibitor-1
1oj5	2.21	Kd = 0.8uM	6.09691	2004	nuclear coactivator 1 (NCoA-1) PAS-B domain
1op9	1.86	Kd = 0.7nM	9.15	2003	human lysozyme
1oph	2.3	Kd = 5nM	8.3	2003	S195A TRYPSIN
1opi	NMR	Kd = 50nM	7.30103	2004	the C-terminal RRM (RRM3) of human U2AF65
1oqp	NMR	Kd = 50nM	7.30103	2003	Chlamydomonas reinhardtii caltractin (CRC-C)
1otr	NMR	Kd = 155uM	3.81	2003	CUE2-1
1oxg	2.2	Ki = 0.027nM	10.56864	2004	Chymotrypsinogen A
1oyh	2.62	Kd = 42nM	7.376751	2004	Antithrombin-III E381A mutant
1ozs	NMR	Kd = 31uM	4.508638	2003	cCTnC.2Ca2+
1p4u	2.2	Kd = 5uM	5.30103	2003	¬¶√É-adaptin ear domain of GGA3
1p69	3.1	Kd = 76nM	7.119186	2004	coxsackievirus and adenovirus receptor domain 1(CAR D1)
1p6a	2.9	Kd = 35nM	7.455932	2004	coxsackievirus and adenovirus receptor domain 1(CAR D1)
1p9d	NMR	Kd = 12.9uM	4.89	2003	S5a-UIM-2
1pd7	NMR	Kd = 0.3uM	6.522879	2004	PAH2 domain of mSin3B, PAH2B
1pdq	1.76	Kd =	5.30103	2003	Polycomb protein

		5uM			
1pjm	2.5	Kd = 180nM	6.744728	2003	mouse Importin-alpha
1pjn	2.5	Kd = 22nM	7.657578	2003	mouse Importin-alpha
1pmx	NMR	IC50 = 7.2uM	5.142667	2003	Insulin-like growth factor IB(IGF-I)
1ppe	2	Kd = 3pM	11.52	1994	Bovine trypsin
1q0w	NMR	Kd = 277uM	3.55752	2003	Vps27 UIM-1
1q5w	NMR	Kd = 126uM	3.9	2004	Ubiquitin
1q68	NMR	Kd = 400nM	6.39794	2003	T cell coreceptors CD4
1q69	NMR	Kd = 900nM	6.045757	2003	T cell coreceptors CD8-alpha
1qng	2.1	Kd = 13nM	7.89	2000	cyclophilin
1qwe	NMR	Kd = 1.2uM	5.92	1996	tyrosine-protein kinase transforming protein sr
1qwf	NMR	Kd = 0.45uM	6.35	1996	tyrosine-protein kinase transforming protein sr
1r0r	1.1	Kd = 29.4pM	10.53	2003	Serine Protease Subtilisin Carlsberg
1r8u	NMR	Kd = 13nM	7.89	2004	CITED2
1rgj	NMR	IC50 = 4.9nM	8.309804	2003	¬¶√Å-Bungarotoxin
1ri8	1.85	Kd = 2.9nM	8.54	2005	Hen Egg White Lysozyme
1rjc	1.4	Kd = 77pM	10.11	2005	Hen Egg White Lysozyme
1rkc	2.7	Kd = 39nM	7.408936	2004	vinculin head domain

1rke	2.35	Kd = 50nM	7.3	2004	vinculin head (1-258)
1ry7	3.2	Kd = 0.23uM	6.64	2004	3 Ig form of FGFR3c
1s3k	1.9	Kd = 0.24uM	6.62	2004	Lewis Y Tetrasaccharide
1s5q	NMR	Kd = 5.2uM	5.283997	2004	mammalian mSin3A (mSin3A) PAH2 domain
1sb0	NMR	Kd = 15uM	4.823909	2004	KIX Domain of CBP
1shy	3.22	Kd = 90nM	7.05	2004	Met Receptor(C604S)
1smf	2.1	Ki = 0.12uM	6.92	1994	trypsin
1sq0	2.6	Kd = 30nM	7.52	2004	Glycoprotein Ib alpha
1syq	2.42	Kd = 14.7nM	7.832683	2004	vinculin head (Vh) domain
1t01	2.06	Kd = 39nM	7.408936	2004	vinculin head domain vh
1t0p	1.66	Kd = 25uM	4.6	2005	ICAM-3
1t44	2	Kd = 1uM	6	2004	chimera of gelsolin domain 1 and c-terminal
1t5z	2.3	Kd = 33uM	4.48	2005	androgen receptor
1t63	2.07	Kd = 15uM	4.82	2005	androgen receptor
1t6b	2.5	Kd = 0.4nM	9.4	2004	Anthrax protective antigen
1t79	1.8	Kd = 920nM	6.04	2004	androgen receptor
1t7f	1.6	Kd = 1.8uM	5.74	2004	androgen receptor
1t7r	1.4	Kd =	5.96	2004	androgen receptor

		1.1uM			
1ta3	1.7	Ki = 9nM	8.05	2004	xylanase inhibitor protein I
1taw	1.8	Ki = 0.02nM	10.7	1997	BOVINE TRYPSIN
1tba	NMR	Kd = 1nM	9	1999	TAFII230
1tdq	2.6	Kd = 12nM	7.920819	2004	rat aggrecan CLD
1te1	2.5	Ki = 3.4nM	8.47	2004	xylanase inhibitor protein I
1tlh	NMR	Kd = 300uM	3.522879	2004	Bacteriophage T4 AsiA
1tm1	1.7	Ki = 0.003nM	11.52	2004	subtilisin bpn' precursor
1tm3	1.57	Ki = 0.019nM	10.72	2004	subtilisin bpn' precursor
1tm4	1.7	Ki = 0.13nM	9.89	2004	subtilisin bpn' precursor
1tm5	1.45	Ki = 0.017nM	10.77	2004	subtilisin bpn
1tm7	1.59	Ki = 0.0033nM	11.48	2004	subtilisin bpn
1to1	1.68	Ki = 0.46nM	9.34	2004	subtilisin bpn
1u0i	NMR	Kd = 70nM	7.154902	2004	IAAL-E3
1u0s	1.9	Kd = 0.23uM	6.64	2004	CheA P2 domain
1u5s	NMR	Kd = 3mM	2.52	2005	Nck-2 SH3 domain
1uel	NMR	Kd = 3.4uM	5.47	2004	UIM wild type S5a
1ugh	1.9	IC50 =	10.92	1999	Human UDG

		12pM			
1uj0	1.7	Kd = 27uM	4.568636	2003	STAM2 SH3 domain
1ukh	2.35	Kd = 0.42uM	6.376751	2004	Mitogen-activated protein kinase 8 isoform 4
1upk	1.85	Kd = 400nM	6.39794	2004	human protein 25¬¶√Å (MO25¬¶√Å)
1us7	2.3	Kd = 3.37uM	5.47	2004	N-HSP90(1-214)
1usu	2.15	Kd = 3.3uM	5.48	2004	M-HSP90
1uti	1.5	Kd = 2.4uM	5.619789	2004	Grb2-related adaptor protein 2(SH3 domain)
1v18	2.1	Kd = 10nM	8	2005	BETA-CATENIN(134-671)
1vet	1.9	Kd = 12.8nM	7.89279	2004	Mitogen-activated protein kinase kinase 1 interacting protein 1, p14
1veu	2.15	Kd = 12.8nM	7.89	2004	p14
1vg0	2.2	Kd = 5nM	8.3	2004	rab proteins geranylgeranyltransferase componen
1vwf	1.92	Kd = 310nM	6.51	1998	streptavidin
1wa7	NMR	Kd = 9.58uM	5.018634	2005	TYROSINE-PROTEIN KINASE LYN SH3 Domain
1wa8	NMR	Kd = 11nM	7.958607	2005	CFP-10
1wlp	NMR	Kd = 0.64uM	6.19382	2005	tandem SH3 domains of p47phox
1wpx	2.7	Ki = 1.80nM	8.74	2005	carboxypeptidase Y
1wq1	2.5	Kd = 17uM	4.77	1998	Ras GTPase
1wqj	1.6	Kd = 865nM	6.06	2005	NBP-4(3-82)

1wr1	NMR	Kd = 14.8uM	4.83	2005	Dsk2p UBA
1wrd	1.75	Kd = 409uM	3.39	2005	Tom1 GAT domain
1x8s	2.5	Kd = 8uM	5.09691	2004	Par-6 PDZ domain
1xb7	2.5	Kd = 0.6uM	6.221849	2004	the ligand binding domain (LBD) of the estrogen-related receptor ¬¶√Å (ERR¬¶√Å, NR3B1)
1xdt	2.65	Kd = 10nM	8	1998	DIPHTHERIA TOXIN
1xg2	1.9	Kd = 5nM	8.3	2005	pectin methylesterase
1xj7	2.7	Kd = 39uM	4.41	2005	androgen receptor
1xr0	NMR	Kd = 10uM	5	2004	Basic fibroblast growth factor receptor 1
1xt9	2.2	Kd = 200nM	6.7	2004	human Nedd8-specific protease, Den1
1y1k	1.56	Ki = 0.3nM	9.52	2005	subtilisin bpn
1y2a	2.2	Kd = 45.8nM	7.339135	2005	Importin ¬¶√Å-2 Subunit
1y33	1.8	Ki = 1.7nM	8.77	2005	subtilisin bpn
1y34	1.55	Ki = 0.52nM	9.28	2005	subtilisin bpn
1y3b	1.8	Ki = 0.31nM	9.51	2005	subtilisin bpn
1y3c	1.69	Ki = 0.025nM	10.6	2005	subtilisin bpn
1y3d	1.8	Ki = 0.56nM	9.25	2005	subtilisin bpn
1y48	1.84	Ki = 1.7nM	8.77	2005	subtilisin bpn

1y4a	1.6	Ki = 1.2nM	8.92	2005	subtilisin bpn
1y4d	2	Ki = 1.2nM	8.92	2005	subtilisin bpn
1y6k	2.52	Kd = 1nM	9	2005	human Interleukin-10, IL-10
1y6m	2.8	Kd = 173nM	6.76	2005	soluble IL-10R1 chain
1y6n	2.7	Kd = 27nM	7.57	2005	soluble IL-10R1 chain
1y8n	2.6	Kd = 1.17uM	5.93	2005	pyruvate dehydrogenase [lipoamide] kinase
1yc0	2.6	IC50 = 4nM	8.39794	2005	Hepatocyte growth factor activator (HGFA)
1ycs	2.2	Kd = 30nM	7.52	1997	P53 core domain
1ydi	1.8	Kd = 1.78nM	8.74958	2005	vinculin N-terminal seven-helical bundle domain (Vh1)
1yrt	2.1	Kd = 1nM	9	2006	wild type CyaA-ACD
1yru	2.5	Kd = 1nM	9	2005	wild type CyaA-ACD
1yvb	2.7	Ki = 6.5nM	8.19	2006	Falcipain 2
1yvh	2.05	Kd = 43nM	7.366531	2005	CBL E3 ubiquitin protein ligase
1ywi	NMR	Kd = 371uM	3.430626	2005	WW domain of Formin-binding protein 11(FBP11)
1yx5	NMR	Kd = 350uM	3.46	2005	S5a UIM-1
1yx6	NMR	Kd = 73uM	4.14	2005	S5a UIM-2
1yy6	1.7	Kd = 0.86uM	6.065502	2005	Ubiquitin carboxyl-terminal hydrolase 7(USP7)
1z92	2.8	Kd =	8	2005	human Interleukin-2

		10nM			
1zgu	NMR	Kd = 98uM	4.01	2006	human Mms2
1zhi	2.7	Kd = 0.2uM	6.7	2005	S.cerevisiae Origin recognition complex subunit 1
1zjd	2.6	IC50 = 1.28nM	8.89	2005	Catalytic Domain of Coagulation Factor XI
1zli	2.09	Ki = 1.3nM	8.89	2005	human carboxypeptidase B
1zsg	NMR	Kd = 7.5uM	5.124939	2005	PIX-SH3
1zub	NMR	Kd = 0.27uM	6.568636	2005	RIM1¬¶√Å PDZ Domain
1zv5	2	Kd = 10nM	8	2006	hen egg white lysozyme
1zvh	1.5	Kd = 70nM	7.15	2006	hen egg white lysozyme
1zvy	1.63	Kd = 0.1nM	10	2006	hen egg white lysozyme
2a0t	NMR	Kd = 15uM	4.823909	2005	Serine/threonine-protein kinase RAD53
2a24	NMR	Kd = 30uM	4.522879	2006	ARNT PAS domain
2a25	2.2	Kd = 24uM	4.619789	2005	Ubiquitin ligase SIAH1
2a3i	1.95	IC50 = 0.9uM	6.05	2005	mineralocorticoid receptor
2a78	1.81	Kd = 60nM	7.22	2005	ras-related protein ral-a
2a7u	NMR	Kd = 120nM	6.920819	2005	the sigma-subunit N-terminal domain
2a9k	1.73	Kd = 60nM	7.22	2005	RalA-GDP
2aq2	1.8	Kd = 11nM	7.96	2006	superantigen SEC3-1D3 mutant

2aq9	1.8	Ki = 50nM	7.30103	2006	Acyl-[acyl-carrier-protein]--UDP-N-acetylglucosamine O-acyltransferase
2arp	2	Kd = 430nM	6.37	2006	Activin A
2asu	1.85	Kd = 1.0nM	9	2005	¬¶√Ç-chain of Hepatocyte growth factor-like protein
2axi	1.4	IC50 = 0.14uM	6.85	2006	ubiquitin-protein ligase e3 mdm2
2b0z	2.7	Kd = 100uM	4	2005	cytochrome c peroxidase
2b12	3.02	Kd = 20uM	4.7	2005	cytochrome c peroxidase
2b42	2.5	Kd = 1.07nM	8.97	2006	bacillus subtilis xylanase A
2b7c	1.8	Kd = 0.4uM	6.4	2006	eEF1A
2b87	NMR	Kd = 100nM	7	2006	ZTaq
2bba	1.65	Kd = 70nM	7.154902	2006	Ephrin type-B receptor 4
2l9s	NMR	Kd = 2.2uM	5.66	2011	PHD finger protein 12 residues 200-241
2nqd	1.75	Ki = 39pM	10.41	2007	human cathepsin L
2nwn	2.15	Ki = 500nM	6.30103	2007	Plasminogen activator, urokinase
2btf	2.55	Kd = 2.3uM	5.64	1994	bovine profilin
2c0l	2.3	Kd = 109nM	6.96	2006	TPR DOMAIN OF HUMAN PEX5P
2c1m	2.2	Kd = 1.1nM	8.96	2005	IMPORTIN-ALPHA2 SUBUNIT
2c7m	2.4	Kd = 6.4uM	5.19	2006	RUZ-MIU
2cpk	2.7	Ki =	8.638272	1993	cAMP-DEPENDENT PROTEIN

		2.3nM			KINASE, CATALYTIC SUBUNIT
2den	NMR	Kd = 17uM	4.77	2006	BMSC-UbP UBA domain(I12A)
2djy	NMR	Kd = 40uM	4.39794	2006	The Smurf2 WW3 Domain
2dsp	2.5	Kd = 3uM	5.522879	2006	insulin-like growth factor IGF1
2dx5	3.35	Kd = 0.33mM	3.481486	2006	mouse Eap45 GLUE
2ez5	NMR	Kd = 3uM	5.522879	2006	E3 ubiquitin-protein ligase NEDD4
2f31	2.1	Kd = 102nM	6.9914	2006	DID domain
2f4m	1.85	Kd = 65nM	7.19	2006	Mouse PNGase core
2fci	NMR	Kd = 70nM	7.154902	2006	C-termainl SH2 domain from phospholipase C-gamma-1 comprising residues 663-759(PLC¬¶√É)
2few	NMR	Kd = 3.7mM	2.43	2006	IIAMtl(H554Q)
2fju	2.2	Kd = 5.3uM	5.28	2006	GTPase Rac1
2flu	1.5	Kd = 20nM	7.69897	2006	Kelch-like ECH-associated protein 1
2ftl	1.62	Kd = 60fM	13.22	2006	trypsin
2ftm	1.65	Kd = 16pM	10.8	2006	trypsin
2fts	2.41	Kd = 90nM	7.045757	2006	The E-domain of rat gephyrin (Geph-E, residues 318¬®C736)
2fuh	NMR	Kd = 300uM	3.52	2006	UbcH5c
2fuu	NMR	Kd = 2.7uM	5.568636	2006	bromodomain PHD finger transcription factor
2fyl	NMR	Kd =	5.55	2006	double module of LRP, CR56

		2.8uM			
2g2u	1.6	Kd = 1.25uM	5.9	2006	SHV-1 Beta-lactamase
2g2w	1.8	Kd = 582nM	6.24	2006	SHV D104K Beta-lactamase
2g6q	2	Kd = 1.5uM	5.823909	2006	mouse Inhibitor of growth protein 2
2g81	1.55	Ki = 13.6nM	7.866461	2007	beta-trypsin
2gng	1.87	IC50 = 6uM	5.221849	2006	Protein kinase A fivefold mutant of Rho-kinase
2gph	1.9	Kd = 5uM	5.30103	2006	Mitogen-activated protein kinase 1
2gww	2.72	Kd = 0.11nM	9.958608	2006	vinculin domain (Vh1)
2h13	1.58	Kd = 4.5uM	5.346787	2006	WD-repeat protein 5(WDR5)
2h2d	1.7	Kd = 4.3uM	5.366531	2006	NAD-dependent deacetylase
2h2g	1.63	Kd = 9.2uM	5.036212	2006	NAD-dependent deacetylase
2h2h	2.2	Kd = 10.5uM	4.978811	2006	NAD-dependent deacetylase
2h9p	1.91	Kd = 192uM	3.717	2006	WD-repeat protein 5(WDR5,àÜ23)
2hd5	1.85	Kd = 2.8uM	5.55	2006	USP2
2hev	2.41	IC50 = 62nM	7.21	2006	human OX40
2hle	2.05	Kd = 40nM	7.39794	2006	EphB4
2hmh	2	Kd = 70nM	7.154902	2006	Suppressor of cytokine signaling 3(SOCS3)
2hqw	1.9	Kd = 2nM	8.69897	2007	calcium saturated CaM

2hrk	2.05	Kd = 9nM	8.05	2006	Glutamyl-t-RNA synthetase (GluRS-N, amino acids 1-207)
2hsq	3.97	Kd = 6.61nM	8.179798	2006	vinculin domain (Vh1)
2hth	2.7	Kd = 105uM	3.98	2006	human EAP45/ESCRT-II GLUE domain
2hug	NMR	Kd = 1.06uM	5.974694	2007	Signal recognition particle 43 kDa protein, chloroplast
2ixq	NMR	Kd = 0.7uM	6.15	2006	PROTEIN AFAD(AfaD)
2j12	1.5	Kd = 20nM	7.7	2006	CAR D1
2jby	2.41	IC50 = 50nM	7.30103	2007	M11L PROTEIN
2jgz	2.9	Kd = 1mM	3	2007	PHOSPHO-CDK2
2jk9	1.79	Kd = 150nM	6.82	2008	SPRY DOMAIN-CONTAINING SOCS BOX PROTEIN 1
2jnw	NMR	Kd = 0.78uM	6.107905	2007	DNA excision repair protein ERCC-1
2jod	NMR	Ki = 350nM	6.46	2007	hPAC1-RS(N-terminal)
2jq9	NMR	Kd = 33.4uM	4.476254	2007	Vacuolar protein sorting-associating protein 4A
2jqi	NMR	Kd = 10uM	5	2008	Rad53 FHA1 domain
2jqk	NMR	Kd = 402uM	3.395774	2007	Vacuolar protein sorting-associating protein 4B
2jql	NMR	Kd = 0.3uM	6.522879	2008	the yeast Dun1 FHA domain
2jt4	NMR	Kd = 40uM	4.4	2007	Sla1 SH3-3
2jti	NMR	Kd = 0.588uM	6.23	2008	yeast cytochrome c peroxidase
2ju0	NMR	Kd =	7	2007	Ca2+ bound Frq1

		100nM			
2jy6	NMR	Kd = 20uM	4.69897	2008	UQ1-UBA domain
2k00	NMR	Kd = 24uM	4.619789	2008	Talin-1
2k2r	NMR	Kd = 1.3mM	2.886057	2008	Alpha-parvin
2k2s	NMR	Kd = 53nM	7.275724	2009	Micronemal protein 1 Galectin-like domain
2k2u	NMR	Kd = 360nM	6.443697	2008	Tfb1 subunit of TFIIH
2k3s	NMR	Kd = 2.7uM	5.568636	2008	CH-domain of the smoothelin-like 1, SMTNL1-CH
2k3u	NMR	Kd = 8.4nM	8.08	2009	immune evasion protein CHIPS
2k3w	NMR	Kd = 5.8uM	5.236572	2008	Vacuolar protein sorting-associating protein 4A
2k42	NMR	Kd = 35nM	7.455932	2008	GTPase Binding Domain of WASP
2k5b	NMR	Kd = 4uM	5.39794	2008	Heat shock protein HSP 90-alpha
2k6d	NMR	Kd = 171uM	3.767004	2008	SH3 domain-containing kinase-binding protein 1, CIN85 SH3-C
2k79	NMR	Kd = 0.67mM	3.173925	2009	interleukin-2 tyrosine kinase SH2 domain
2k7a	NMR	Kd = 0.67mM	3.173925	2009	interleukin-2 tyrosine kinase SH2 domain
2k7l	NMR	Kd = 0.7uM	6.154902	2009	C-terminal domain of human RAP74
2k8b	NMR	Kd = 1.8mM	2.74	2009	PLAA family ubiquitin binding domain (PFUC) cis isomer
2k8c	NMR	Kd = 1.8mM	2.74	2009	PLAA family ubiquitin binding domain (PFUC) trans isomer
2k8f	NMR	Kd = 2.7uM	5.568636	2009	CREB-binding protein (CBP) taz2

2ka4	NMR	Kd = 58nM	7.236572	2009	CREB-binding protein CBP-TAZ1
2ka6	NMR	Kd = 52nM	7.283997	2009	CREB-binding protein CBP-TAZ2
2kbr	NMR	Kd = 25.4uM	4.595166	2009	N terminal domain of Harmonin
2kbw	NMR	Kd = 0.32uM	6.49	2009	core domain of human Mcl-1 protein
2kc8	NMR	Kd = 200nM	6.7	2009	E. coli toxin RelE (R81A/R83A) mutant
2kff	NMR	Kd = 245uM	3.610834	2009	EH domain-containing protein 1 C-terminal domain
2kfg	NMR	Kd = 1.2mM	2.920819	2009	EH domain-containing protein 1 C-terminal domain
2kfh	NMR	Kd = 2.4mM	2.619789	2009	EH domain-containing protein 1 C-terminal domain
2khs	NMR	Kd = 0.36uM	6.443697	2009	Thermonuclease residues 83-203
2kj4	NMR	Kd = 65nM	7.187087	2009	plasminogen kringle 2
2knb	NMR	Kd = 11uM	4.958607	2009	parkin Ubl domain
2knh	NMR	Kd = 7uM	5.15	2009	eTAFH domain of AML1-ETO
2koh	NMR	Kd = 6uM	5.22	2010	mouse Partitioning defective 3 homolog, PDZ 3 domain
2kpl	NMR	Kd = 0.23uM	6.64	2010	second PDZ domain of MAGI-1 (Membrane-associated guanylate kinase)
2krd	NMR	Kd = 2000uM	2.7	2010	Regulatory Domain of Human Cardiac Troponin C and Ca2+
2ks9	NMR	Ki = 9.02mM	2.04	2010	Substance-P receptor
2ksa	NMR	Ki = 871uM	3.06	2010	Substance-P receptor

2ksb	NMR	Ki = 754uM	3.12	2010	Substance-P receptor
2ksp	NMR	Kd = 57uM	4.24	2010	C-terminal domain of EH domain-containing protein 1
2ktf	NMR	Kd = 23uM	4.64	2010	human polymerase iota UBM2
2kup	NMR	Kd = 0.2uM	6.7	2010	PTB domain of SNT-2
2kvm	NMR	Kd = 108.4uM	3.96	2010	Chromobox protein homolog 7, CBX7 chromodomain
2kwi	NMR	Kd = 184nM	6.74	2010	Ras-related protein Ral-B Q72L mutant
2kwj	NMR	Kd = 0.5uM	6.3	2010	human Zinc finger protein DPF3b PHD12
2kwn	NMR	Kd = 7.4uM	5.13	2010	human Zinc finger protein DPF3b PHD12
2kwo	NMR	Kd = 46.9uM	4.33	2010	human Zinc finger protein DPF3b PHD12
2kwu	NMR	Kd = 51uM	4.29	2010	C-terminal ubiquitin-binding motif, UBM2 of murine Polymerase iota
2kwv	NMR	Kd = 90uM	4.05	2010	C-terminal ubiquitin-binding motif, UBM1 of murine Polymerase iota
2kxh	NMR	Kd = 14uM	4.85	2010	FIR RRM1-RRM2
2kxw	NMR	Kd = 10nM	8	2011	C-domain Fragment (UNP Residues 77-149) of apo Calmodulin
2kzu	NMR	Kd = 60uM	4.22	2010	DAXX N-Terminal Helical Bundle Domain (55-144)
2l0f	NMR	Kd = 172uM	3.76	2010	human polymerase iota UBM2 (P692A mutant)
2l0i	NMR	Kd = 15uM	4.82	2010	Rtt103 CTD-interacting domain
2l1l	NMR	Kd = 7nM	8.15	2011	Exportin-1 CRM1 and RanGTP
2l6e	NMR	Kd =	6	2010	Capsid protein p24(W184A, M185A)

		1uM			
2nm1	2.15	Kd = 34nM	7.468521	2006	Botulinum neurotoxin type B(BoNT)
2o3b	2.3	Ki = 3.2pM	11.49	2006	Nuclease A
2o8v	3	Ki = 1.1uM	5.96	2007	PAPS reductase
2o9v	1.63	Kd = 288uM	3.540607	2007	Ponsin
2oi3	NMR	Kd = 0.23uM	6.638272	2007	Tyrosine-protein kinase HCK
2omt	2	Kd = 100nM	7	2007	HEC1
2omu	1.8	Kd = 0.6nM	9.22	2007	HEC1
2omv	1.9	Kd = 1.2nM	8.920818	2007	Internalin-A S192N Y369S mutant
2omw	1.85	Kd = 10uM	5	2007	Internalin-A S192N Y369S mutant
2omx	1.7	Kd = 120nM	6.92	2007	HEC1
2omy	1.7	Kd = 0.2uM	6.69897	2007	Internalin-A S192N mutant
2omz	1.6	Kd = 400nM	6.4	2007	HEC1
2oob	1.9	Kd = 57uM	4.244125	2007	UBA domain from Cbl-b ubiquitin ligase
2ot3	2.1	Kd = 1.8uM	5.74	2007	rabex-5 VPS9 domain
2oza	2.7	Kd = 2.5nM	8.6	2007	p38alpha MAPK
2p43	1.65	Kd = 23nM	7.638272	2008	RNase A
2p44	1.8	Kd = 20nM	7.69897	2008	RNase A

2p45	1.1	Kd = 116nM	6.935542	2008	RNase A
2p47	2.5	Kd = 116nM	8.936	2008	RNase A
2p48	2.3	Kd = 116nM	8.936	2008	RNase A
2p49	1.38	Kd = 23nM	7.638272	2007	RNASE A
2p8q	2.35	Kd = 15.6nM	7.806875	2008	importin beta
2r05	2.55	Kd = 7uM	5.154902	2007	ALIX
2pon	NMR	Kd = 1.4uM	5.853872	2007	Apoptosis regulator Bcl-xL
2pr9	2.51	Kd = 42.2nM	7.374688	2008	AP-2 complex subunit mu-1
2ptc	1.9	Kd = 60fM	13.22	1983	beta-trypsin
2ptt	1.63	Kd = 4uM	5.39794	2007	NK cell receptor 2B4 (CD244)
2px9	NMR	Kd = 87.4uM	4.058488	2007	SUMO-activating enzyme subunit 2
2qc1	1.94	Kd = 10pM	11	2007	mouse nicotinic acetylcholine receptor (nAChR) a1 subunit
2qic	2.1	Kd = 3.3uM	5.481486	2008	Inhibitor of growth protein 1
2qna	2.84	Kd = 83nM	7.080922	2008	Imp beta (127-876)
2qur	2.5	IC50 = 500nM	6.30103	2008	F327A/K285P Mutant of cAMP-dependent Protein Kinase
2qxv	1.82	Kd = 0.38uM	6.420217	2007	Embryonic ectoderm development, EED
2r02	2.6	Kd = 8uM	5.09691	2007	ALIX
2rmk	NMR	Kd =	6.769551	2007	HR1b

		170nM			
2rms	NMR	Kd = 134nM	6.872895	2008	mSin3A PAH1
2rnr	NMR	Kd = 150nM	6.823909	2008	hTFIIEa AC-D
2rnw	NMR	Kd = 1051uM	2.978397	2008	Histone acetyltransferase PCAF
2rnx	NMR	Kd = 402uM	3.395774	2008	Histone acetyltransferase PCAF
2rol	NMR	Kd = 24uM	4.619789	2009	Epidermal growth factor receptor kinase substrate 8-like protein 1 SH3 domain
2roz	NMR	Kd = 0.32uM	6.49485	2008	C-terminal PID Domain of Fe65L1
2rr3	NMR	Kd = 2.1uM	5.68	2010	human Vesicle-associated membrane protein-associated protein A, VAP-A MSP domain
2sic	1.8	Kd = 71.2pM	10.15	1993	Subtilisin Bpn
2sni	2.1	Kd = 2pM	11.7	1988	SUBTILISIN NOVO
2tgp	1.9	Kd = 2.4uM	5.62	1983	TRYPSINOGEN
2uuy	1.15	Ki = 5.6nM	8.25	2007	Bovine Cationic Trypsin
2uyz	1.4	Kd = 82nM	7.09	2007	UBC9
2v3b	2.45	Kd = 5uM	5.3	2007	RUBREDOXIN REDUCTASE
2v4z	2.8	Kd = 1.25uM	5.9	2008	HUMAN G-PROTEIN SUBUNIT ALPHA (GNAI3)
2v52	1.45	Kd = 1.9uM	5.721246	2008	G-actin
2v6x	1.98	Kd = 28uM	4.552842	2007	Vps4 MIT-domain

2v8s	2.22	Kd = 22uM	4.66	2007	Vti1b
2v9t	1.7	Kd = 8.2nM	8.09	2007	Robo1 Ig
2vay	1.94	Kd = 7.9nM	8.1	2008	CALMODULIN
2vda	NMR	Kd = 3uM	5.522879	2007	Translocase subunit SecA
2vdb	2.52	Kd = 0.15nM	9.82	2008	human serum albumin (HSA)
2ver	NMR	Kd = 13.1uM	4.882729	2008	N-CEA
2vln	1.6	Kd = 1.68pM	11.77469	2008	E9 DNase N75A
2vlo	1.8	Kd = 0.892pM	12.04963	2008	E9 DNase K97A
2vlp	2	Kd = 0.543pM	12.2652	2008	E9 DNase R54A
2vlq	1.6	Kd = 22.8pM	10.64207	2008	E9 DNase F86A
2vog	1.9	Kd = 210nM	6.677781	2008	mouse Prosurvival Protein A1
2voh	1.9	Kd = 1nM	9	2008	mouse Prosurvival Protein A1
2voi	2.1	Kd = 1.1nM	8.958608	2008	mouse Prosurvival Protein A1
2vsm	1.8	Kd = 35nM	7.455932	2008	NIPAH VIRUS ATTACHMENT GLYCOPROTEIN
2vwf	1.58	Kd = 8.7uM	5.060481	2009	GROWTH FACTOR RECEPTOR-BOUND PROTEIN 2 SH3 DOMAIN
2w85	NMR	Kd = 9.17uM	5.037631	2009	Peroxisomal Membrane Anchor Protein Pex14
2w9r	1.7	Kd = 4.8uM	5.318759	2009	ATP-Dependent Clp Protease Adapter Protein ClpS
2wel	1.9	Kd =	6.22	2009	CALCIUM/CALMODULIN-DEPENDENT PROTEIN KINASE

		0.6uM			TYPE II DELTA CHAIN
2wfj	0.75	Kd = 70nM	7.154902	2009	Peptidyl-prolyl cis-trans isomerase G
2wfx	3.2	Kd = 14nM	7.853872	2009	HUMAN HEDGEHOG-INTERACTING PROTEIN HIP
2wg4	3.15	Kd = 73.9nM	7.131356	2009	HUMAN HEDGEHOG-INTERACTING PROTEIN HIP
2wh6	1.5	Kd = 18nM	7.74	2010	BHRF1 PROTEIN
3bzd	2.3	Kd = 96uM	4.02	2009	T cell receptor beta chain 8.2 Variable domain
2wo2	2.45	Kd = 10.8uM	4.966576	2009	EPHRIN TYPE-A RECEPTOR
2wo3	2.35	Kd = 2.3uM	5.638272	2009	EPHRIN TYPE-A RECEPTOR
2wp3	1.48	Kd = 0.794uM	6.1	2010	TITIN M10
2wpt	1.78	Kd = 0.1uM	7	2010	COLICIN-E9
2wwk	1.7	Kd = 1.28uM	5.89	2010	TITIN M10
2wwx	1.5	Kd = 79nM	7.102373	2009	RAS-RELATED PROTEIN RAB-1 GTPASE DOMAIN
2wy8	1.7	Kd = 360nM	6.44	2010	COMPLEMENT FRAGMENT C3D
2x1x	3.1	Kd = 16nM	7.8	2010	VASCULAR ENDOTHELIAL GROWTH FACTOR RECEPTOR 2, IG-LIKE DOMAINS 2 AND 3
2xgy	1.8	Kd = 30uM	4.52	2010	RELIK CAPSID N-TERMINAL DOMAIN
2xpx	2.05	Kd = 150nM	6.82	2011	APOPTOSIS REGULATOR BHRF1
2xs8	2.5	Kd = 24.5uM	4.61	2010	ALIX Bro1-V domains (KK268, 269YY mutant)
2xtt	0.93	Ki =	10.7	2010	CATIONIC TRYPSIN

		20pM			
2y9m	2.6	Kd = 2nM	8.7	2011	UBIQUITIN-CONJUGATING ENZYME E2-21 KDA, Pex4p UBC domain(15-183)
2z58	1.88	Kd = 16nM	7.79588	2008	G56W-propeptide
2z7f	1.7	IC50 = 13nM	7.886056	2008	human neutrophil elastase
3ajb	2.5	Kd = 40.8nM	7.39	2010	human Peroxisomal biogenesis factor 3
3alz	4.51	Kd = 0.52uM	6.28	2011	measles virus hemagglutinin (MV-H)
3aon	2	Kd = 3.2nM	8.49	2011	prokaryotic V-ATPase of Enterococcus hirae, D subunit
3beg	2.9	Kd = 50nM	7.30103	2008	SRPK1
3bh6	2.6	Kd = 95nM	7.022276	2008	Arl3-mGppNHp
3blh	2.48	Kd = 300nM	6.522879	2008	Cell division protein kinase 9, CDK9
3bn3	2.1	Kd = 20uM	4.69897	2008	Intercellular adhesion molecule 5, ICAM-5
3bs5	2	Kd = 92.5nM	7.033858	2008	SAM domain of CNKin
3btr	2.6	Kd = 5uM	5.30103	2008	Importin subunit alpha-2
3bu6	1.95	Kd = 1.3uM	5.886056	2008	insulin receptor subunit beta
3bum	2	Kd = 0.32uM	6.49485	2008	E3 ubiquitin-protein ligase CBL
3bun	2	Kd = 0.61uM	6.21467	2008	E3 ubiquitin-protein ligase CBL
3c3o	2.15	Kd = 44uM	4.356547	2008	Programmed cell death 6-interacting protein
3c3q	2.1	Kd =	4.318759	2008	Programmed cell death 6-interacting

		48uM			protein
3c3r	2.02	Kd = 41uM	4.387216	2008	Programmed cell death 6-interacting protein
3c4o	1.7	Kd = 4.6nM	8.337242	2008	Beta-lactamase SHV-1
3c4p	1.75	Kd = 4.4nM	8.356547	2008	Beta-lactamase SHV-1
3c59	2.3	IC50 = 5nM	8.30103	2008	Glucagon-like peptide 1 receptor
3c5t	2.1	IC50 = 0.6nM	9.221848	2008	Glucagon-like peptide 1 receptor
3cfs	2.4	Kd = 1.1uM	5.958607	2008	Histone-binding protein RBBP7
3ch5	2.1	Kd = 120uM	3.920819	2008	RanGDP
3cqc	2.53	Kd = 4nM	8.39794	2008	human Nucleoporin Nup107
3cqg	3	Kd = 4nM	8.39794	2008	human Nucleoporin Nup107
3cs8	2.3	IC50 = 1.7uM	5.769551	2008	PPARgama
3cx6	2.5	Kd = 4.8uM	5.318759	2008	Guanine nucleotide-binding protein alpha-13 subunit Bound to GDP
3cx7	2.25	Kd = 520nM	6.283997	2008	Guanine nucleotide-binding protein alpha-13 subunit Bound to ALF and GDP
3cx8	2	Kd = 340nM	6.468521	2008	Guanine nucleotide-binding protein alpha-13 subunit Bound to GTP gamma S
3d7t	2.9	Kd = 70uM	4.154902	2008	CskKD
3ddc	1.8	Kd = 0.77uM	6.113509	2008	Ras D30E/E31K
3di3	2.9	Kd = 21nM	7.677781	2009	glycosylated human interleukin-7 receptor alpha ectodomain

3doe	2.25	Kd = 20nM	7.7	2009	ADP-ribosylation factor-like protein 2, ARL2
3dow	2.3	Kd = 11.5uM	4.939302	2009	Gamma-aminobutyric acid receptor-associated protein
3ds0	1.6	Kd = 14.9uM	4.826814	2008	HIV-1 Capsid Protein(N183A)
3ds1	1.6	Kd = 2.0uM	5.69897	2008	HIV-1 Capsid Protein(E187A)
3dvm	2.6	Kd = 4.32nM	8.364516	2008	Ca2+/CaM, Calmodulin
3e1z	1.86	Ki = 0.036nM	10.4437	2009	a classic family C1 cysteine protease, papain
3mca	2.74	Kd = 0.39uM	6.41	2010	Elongation factor 1 alpha-like protein, Hsb1
3me2	2.8	Kd = 68pM	10.17	2010	Tumor necrosis factor receptor superfamily member 11A
3oiq	2.4	Kd = 3.8uM	5.42	2010	yeast telomere protein Cdc13 OB1
3eba	1.85	Kd = 2.36nM	8.627088	2008	human Lysozyme C
3eg6	1.72	Kd = 1.7uM	5.769551	2008	WD repeat-containing protein 5
3emh	1.37	Kd = 140nM	6.853872	2008	WD repeat-containing protein 5
3eqs	1.65	Kd = 3.3nM	8.481486	2009	E3 ubiquitin-protein ligase Mdm2
3er5	1.8	Ki = 1nM	9	1991	endothiapepsin
3eu7	2.2	Kd = 0.66uM	6.180456	2009	Partner and localizer of BRCA2
3f1p	1.17	Kd = 120uM	3.920819	2009	Endothelial PAS domain-containing protein 1, HIF2alpha C-terminal PAS domain
3fhc	2.8	Kd = 21.6nM	7.665546	2009	Nuclear pore complex protein Nup214(68-302)

3fii	2.17	Ki = 1.9nM	8.721247	2009	Clostridium botulinum neurotoxin serotype F catalytic domain
3fju	1.6	Ki = 1.6nM	8.79588	2008	human carboxypeptidase A1
3fp6	1.49	Kd = 0.1pM	13	2009	Anionic trypsin-2
3fpu	1.76	Kd = 0.12nM	9.92	2010	Evasin-1
3g7a	2.8	Ki = 9nM	8.045757	2009	HIV gp41 NHR domain
3g7l	2.2	Kd = 0.19uM	6.721246	2009	Chromo domain-containing protein 1, Chp1
3gb8	2.9	Kd = 6.5uM	5.187087	2009	Exportin-1
3gbq	NMR	Kd = 3.5uM	5.455932	1997	Grb2(N-terminal SH3 domain)
3gc3	2.2	Kd = 2.1uM	5.68	2009	Arrestin2S-(1-385)
3gj3	1.79	Kd = 49uM	4.309804	2009	GTP-binding nuclear protein Ran
3gj6	2.7	Kd = 6.5uM	5.187087	2009	GTP-binding nuclear protein Ran
3gni	2.35	Kd = 12nM	7.920819	2009	Protein Mo25 alpha
3gqi	2.5	Kd = 33nM	7.481486	2009	activated tyrosine kinase domain of FGFR1
3gty	3.4	Kd = 0.47nM	9.327902	2009	Trigger factor
3gxu	2.5	Kd = 203nM	6.69	2009	Ephrin type-A receptor 4
3h1z	1.83	Kd = 22uM	4.66	2009	clathrin adaptor AP-2 complex subunit beta-1
3h8k	1.8	Kd = 21nM	7.677781	2009	Ubiquitin-conjugating enzyme E2 G2
3hct	2.1	Kd =	5.829738	2009	TNF receptor-associated factor 6 RING and Zinc Finger 1, TRAF6

		1.48uM			RZ123
3hqh	2.3	Kd = 63.6uM	4.196543	2009	Speckle-type POZ protein MATHx
3hs8	1.9	Kd = 5uM	5.3	2010	Adaptor protein complex AP-2, alpha 2 subunit
3hs9	2.15	Kd = 490nM	6.31	2010	Adaptor protein complex AP-2, subunit beta-1
3i5r	1.7	Kd = 40uM	4.4	2010	phosphatidylinositol 3-kinase (PI3K) SH3 domain
3idb	1.62	Kd = 11.3nM	7.946921	2009	C Holoenzyme of cAMP-dependent Protein Kinase
3idc	2.7	Kd = 0.6nM	9.221848	2009	C Holoenzyme of cAMP-dependent Protein Kinase
3iiw	1.8	Kd = 36.4uM	4.438899	2009	Polycomb protein EED residues 77-441
3iiy	2.65	Kd = 16uM	4.79588	2009	Polycomb protein EED residues 77-441
3ij0	2.45	Kd = 13.2uM	4.879426	2009	Polycomb protein EED residues 77-441
3ij1	2.1	Kd = 20uM	4.69897	2009	Polycomb protein EED residues 77-441
3iol	2.1	IC50 = 500nM	6.30103	2009	Glucagon-like peptide 1 receptor N-terminal extracellular domain
3iqq	2.01	Kd = 2.9uM	5.54	2010	Protein S100-B
3ixe	1.9	IC50 = 2.3uM	5.638272	2009	Integrin-linked protein kinase Ankyrin repeat domain
3jpx	2.05	Kd = 235uM	3.63	2009	Polycomb protein EED
3jza	1.8	Kd = 3pM	11.52	2010	human Ras-related protein Rab-1B
3jzo	1.8	Kd = 3nM	8.52	2009	Human Protein Mdm4
3jzp	1.74	IC50 = 250nM	6.6	2009	Human Protein Mdm4

3jzr	2.1	IC50 = 36nM	7.44	2009	E3 ubiquitin-protein ligase Mdm2
3jzs	1.78	IC50 = 8nM	8.1	2009	E3 ubiquitin-protein ligase Mdm2
3k1r	2.3	Kd = 1nM	9	2010	harmonin NPDZ1 domain
3k26	1.58	Kd = 400uM	3.4	2009	Polycomb protein EED
3k8p	2.6	Kd = 10nM	8	2009	Protein transport protein SEC39
3kj0	1.7	Kd = 2nM	8.7	2010	Induced myeloid leukemia cell differentiation protein Mcl-1
3kj1	1.95	Kd = 2nM	8.7	2010	Induced myeloid leukemia cell differentiation protein Mcl-1
3kj2	2.35	Kd = 2nM	8.7	2010	Induced myeloid leukemia cell differentiation protein Mcl-1
3knb	1.4	Kd = 0.94uM	6.03	2009	titin C-terminus M10
3kuc	1.92	Kd = 0.442uM	6.35	2010	Ras-related protein Rap-1A(E30D/K31E)GDP
3kud	2.15	Kd = 1.7uM	5.77	2010	Ras-related protein Rap-1A GDP
3kv4	2.19	Kd = 1uM	6	2009	PHD finger protein 8, PHF8
3kw5	2.83	Kd = 385nM	6.41	2010	ubiquitin carboxy terminal hydrolase L1
3kyi	2.8	Kd = 218uM	3.66	2010	phosphorylated P1 domain of CheA3
3l3x	1.55	IC50 = 0.71uM	6.15	2010	DHT-bound androgen receptor LBD
3l3z	2	IC50 = 13.6uM	4.87	2010	DHT-bound androgen receptor LBD
3l6x	2.4	Kd = 40uM	4.4	2010	p120 catenin isoform 4A delta 1 (deletion, residues 613-643)
3l9j	2.1	Kd =	9.47	2010	human TNF alpha

		0.34nM			
3lbx	2.8	Kd = 400nM	6.4	2010	human spectrin alpha 0-1
3lms	2.5	Ki = 1.2nM	8.92	2010	human TAFIa
3m18	1.95	Kd = 180pM	9.74	2010	variable lymphocyte receptor VLRA.R2.1
3mj7	2.8	Kd = 5uM	5.3	2010	Junctional adhesion molecule-like, JAML
3mzg	2.1	Kd = 4.97mM	2.3	2010	WT hPRL antagonist, human prolactin receptor antagonist
3mzw	2.9	Kd = 22pM	10.66	2010	Tyrosine kinase-type cell surface receptor HER2 Extracellular domain
3n00	2.6	IC50 = 0.22uM	6.66	2010	truncated human Rev-erba ligand binding domain
3n06	2	Kd = 3.1mM	2.51	2010	WT hPRL antagonist, human prolactin receptor antagonist H27A mutant
3n0p	2.1	Kd = 5.1mM	2.29	2010	WT hPRL antagonist, human prolactin receptor antagonist H30A mutant
3n4i	1.56	Kd = 1.1nM	8.96	2011	SHV-1 D104E beta-lactamase
3ncb	2.1	Kd = 93mM	1.03	2010	WT hPRL antagonist, human prolactin receptor antagonist H180A mutant
3ncc	2.5	Kd = 212mM	0.67	2010	WT hPRL antagonist, human prolactin receptor antagonist
3nvn	2.26	Kd = 9.4nM	8.03	2010	viral-derived Sema7A mimic, A39R
3o34	1.9	Kd = 8.8uM	5.06	2010	Transcription intermediary factor 1-alpha(TRIM24 PHD-Bromo)
3o40	2.1	Ki = 1.4nM	8.85	2011	gp41-5 protein
3o43	2.8	Ki = 9nM	8.05	2011	gp41-5 protein

3oap	2.05	Kd = 0.55uM	6.26	2010	Retinoic acid receptor RXR-alpha(hRXRalpha-LBD)
3ob1	2.2	Kd = 3.83uM	5.42	2010	E3 ubiquitin-protein ligase CBL c-Cbl TKB domain
3oe0	2.9	Ki = 2.5nM	8.6	2010	C-X-C chemokine receptor type 4, Lysozyme Chimera(CXCR4-3)
3ohm	2.7	Kd = 200nM	6.7	2010	Guanine nucleotide-binding protein G(q) subunit alpha
3oky	2.19	Kd = 1.3uM	5.89	2010	Semaphorin-6A
3ol2	2.99	Kd = 5.5uM	5.26	2010	Semaphorin-4D
3olm	2.5	Kd = 90.6uM	4.04	2011	WW3 and HECT domain of E3 ubiquitin-protein ligase RSP5
3ona	2.6	Kd = 0.68uM	6.17	2011	Tumour necrosis factor receptor SECRET domain
3ouw	2.91	Kd = 23nM	7.64	2010	Catenin beta-1
3oux	2.4	Kd = 35nM	7.46	2010	Catenin beta-1
3p71	2.7	Kd = 0.38uM	6.42	2011	Leucine carboxyl methyltransferase 1, LCMT-1
3p92	1.6	Ki = 5.9nM	8.23	2011	Human mesotrypsin
3p95	1.3	Ki = 1.5uM	5.82	2011	Human mesotrypsin
3pnr	2.6	Ki = 11.9nM	7.92	2011	falcipain-2, an important hemoglobinase of Plasmodium
3qc8	2.2	Kd = 11.2uM	4.95	2011	Transitional endoplasmic reticulum ATPase p97/VCP N Domain
3qq8	2	Kd = 1.5uM	5.82	2011	Transitional endoplasmic reticulum ATPase p97-N
3qsk	1.75	Kd = 91nM	7.04	2011	Ribonuclease pancreatic, RNAse A
3rgf	2.2	Kd =	8.15	2011	human Cyclin-dependent kinase 8,

		7.05nM			CDK8
3sgb	1.8	Kd = 17.9pM	10.75	1983	PROTEINASE B (SGPB)
3tgk	1.7	Ki = 12uM	4.920819	2001	delta I16V17/D194N trypsinogen
3t04	2.1	Kd = 52nM	7.28	2011	Tyrosine-protein kinase ABL1 SH2 domain
3tac	2.2	Kd = 0.55uM	6.26	2011	Peripheral plasma membrane protein CASK
3tnf	2.5	Kd < 0.01nM	11	2011	LidA
3uyo	1.83	Kd = 6.8uM	5.17	2011	Tyrosine-protein kinase ABL1 SH2 domain
3zyi	2.6	Kd = 7.3nM	8.14	2011	NetrinG ligands (NGLs) NGL2
4er4	2.1	Ki = 160nM	6.8	1991	endothiapepsin

Table S2. Protein-protein affinity benchmark data set used in the present work.

PDB:chain	Protein A	Protein B	Pubmed	Kd (M)	Method	Temperature (C)	pH
1A2K_C:AB	*Ran GTPase-GDP*	Nuclear transport factor 2	10681579	1.50E-07	ITC	25	7.5
1ACB_E:I	Chymotrypsin	Eglin C	3071573	2.00E-10	Spectrophotometric inhibition assay	21	8
1AHW_AB:C	Fab 5g9	Tissue factor	9480775	3.40E-09	Competitive Inhibition assay	room temp.	not stated
1AK4_A:D	Cyclophilin	HIV capsid	9223641	1.60E-05	ITC	20	6.5
1AKJ_AB:DE	MHC class 1 HLA-A2	T-cell CD8 coreceptor	10072074	0.000126	SPR	25	7.4
1ATN_A:D	Actin	Dnase I	6244947	2.00E-09	Spectrophotometric inhibition assay	30	8
1AVX_A:B	Porcine trypsin	Soybean trypsin inhibitor		4.80E-10	Potentiometric	20	8.3
1AVZ_B:C	HIV-1-NEF protein	Fyn kinase SH3 domain	9778343	1.58E-05	ITC	25	7.5
1AY7_A:B	Rnase SA	Barstar		2.00E-10	Fluorescence inhibition assay	not stated	8
1B6C_A:B	FKBP binding protein	TGFbeta receptor	11583628	2.80E-07	SPR	25	7.4
1BJ1_HL:VW	Fab - vEGF	vEGF	9753694	3.40E-09	SPR	25	4.8
1BRS_A:D	Barnase	Barstar	8507637	2.00E-13	Fluorescence inhibition assay	25	8
1BUH_A:B	CDK2 kinase	Ckshs1	860131	7.70E-	SPR	not stated	7.4

			0	08			
1BVK_DE:F	Fv Hulys11	HEW lysozyme	1560463	1.40E-08	Stopped-flow inhibition	20	7
1BVN_P:T	Alpha-amylase	Tendamistat	14715318	9.20E-12	SPR	25	not stated
1CBW_ABC:D	Chymotrypsin	BPTI	8784199	1.10E-08	Spectrophotometric inhibition assay	22	8.2
1DE4_AB:CF	Hemochromatosis protein HFE	Transferrin receptor ectodom.	11800564	6.80E-08	SPR	not stated	7.5
1DFJ_E:I	Ribonuclease A	Rnase inhibitor	2271559	5.90E-14	Inhibition assay (indirect-Upa Hydrolysis)	25	6
1DQJ_AB:C	Fab Hyhel63	HEW lysozyme	10828942	2.80E-09	SPR	25	7.5
1E4K_AB:C	FC fragment of human IgG 1	Human FCGR III	11544262	1.70E-06	SPR	25	7.4
1E6E_A:B	Adrenoxin reductase	Adrenoxin	15181009	8.56E-07	SPR	not stated	7.4
1E6J_HL:P	Fab 13B5	HIV-1 capsid protein p24	11080628	2.90E-08	SPR	not stated	7.4
1E96_A:B	Rac GTPase	p67 Phox	11090627	2.70E-06	ITC	18	7
1EAW_A:B	Matriptase	BPTI	17475279	4.97E-11	Fluorescence inhibition assay	room temp.	8.8
1EER_A:BC	Erythropoietin	EPO receptor	12463751	3.70E-12	SPR	25	7.4
1EFN_B:A	HIV-1-NEF protein	Fyn kinase SH3 domain	7588629	3.80E-08	SPR	25	7.4

1EMV_A:B	Colicin E9 nuclease	Im9 immunity protein	7577967	2.40E-14	Stopped-flow fluormetry	25	7
1EWY_A:C	Ferredoxin reductase	Ferredoxin	1910307	3.57E-06	Spectroscopic assay	25	8
1EZU_C:AB	D102N Trypsin	Y69F D70P Ecotin	9642073	8.00E-11	Spectroscopic inhibition assay	25	8
1F34_A:B	Porcine pepsin	Ascaris inhibitor 3	4594130	1.00E-10	Spectroscopic inhibition assay	37	2
1F6M_A:C	Thioredoxin reductase	Thioredoxin 1	19933368	2.70E-06		room temp.	8
1FC2_C:D	Staphylococcus Protein A	Human Fc fragment	7646442	2.25E-08	Stopped-flow fluorescence	25	6
1FFW_A:B	Chemotaxis protein CheY	Chemotaxis protein CheA	8377825	1.35E-06	ITC	28	7.4
1FLE_E:I	Elastase	Elafin	2394696	1.00E-09	Inhibition assay	room temp.	7.5
1FQJ_A:B	Gt-alpha	RGS9	10085118	6.70E-08	Fluorescence spectroscopy	room temp.	8
1FSK_BC:A	Fab - Birch pollen antigen Bet V1	Birch pollen antigen Bet V1		2.40E-10	SPR	25	7.4
1GCQ_B:C	GRB2 C-ter SH3 domain	Vav N-ter SH3 domain	11406576	1.70E-05	SPR	25	7.4
1GL1_A:I	Chymotrypsin	PMP-C (LCMI II)	7592720	2.00E-10	Inhibition assay	25	8
1GLA_G:F	Glycerol Kinase	Glucose specific IIIGlc	9538005	1.10E-05	Spectroscopy	room temp.	7
1GPW_A:B	HISF protein	Amidotransferase HISH		5.00E-09	Fluorescence Titration	25	not stated

1GRN_A:B	CDC42 GTPase	CDC42 GAP	9468490	2.39E-07	Fluorescence Spectroscopy	not stated	8
1GXD_A:C	ProMMP2 type IV collagenase	Metalloproteinase inhibitor 2	9368077	5.20E-09	SPR	25	7.2
1H1V_A:G	Actin	Gelsolin precursor C-term	2836434	2.50E-08	Fluorescence spectroscopy	20	8
1H9D_A:B	Runx1 domain of CBFalpha1	Dimerisation domain of CBF-beta	10984496	4.50E-08	Electrophoretic mobility shift assays	0	7.5
1HCF_AB:X	Neurotrophin-4	TrkB-d5 growth factor receptor	11855816	2.60E-10	SPR	not stated	7.4
1HE8_B:A	Ras GTPase	PIP3 kinase	11136978	3.20E-06	Stopped-flow fluometry	20	7.5
1HIA_AB:I	Kallikrein	Hirustatin	8112345	1.30E-08	Inhibition assay	not stated	not stated
1I2M_A:B	Ran GTPase-GDP	RCC1	7548002	2.50E-12	Stopped-flow fluometry	25	7.4
1I4D_D:AB	Rac GTPase	Arfaptin	11346801	3.00E-06	ITC	22	8.7
1IB1_AB:E	14-3-3 protein	Serotonin N-acteylase	11336675	2.00E-08	Sedimentation equilibrium	4	6.5
1IBR_A:B	Ran GTPase-GDP	Importin beta	10995230	1.00E-09	Fluorescence titration	20	7.4
1IJK_A:BC	Von Willebrand Factor dom. A1	Botrocetin	10713059	2.30E-08	Radioligand binding	room temp.	7.4
1IQD_AB:C	Fab - Factor VIII domain C2	Factor VIII domain C2	9657749	<1.4e-11	SPR	not stated	7.4

1J2J_A:B	Arf1 GTPase.GNP-RanBD1	GAT domain of GGA1	12679809	1.10E-06	SPR	25	8
1JIW_P:I	Alkaline metallo-proteinase	Proteinase inhibitor	10770939	4.00E-12	Inhibition assay	25	7
1JMO_A:HL	Heparin cofactor	Thrombin	9162031	1.15E-07	Inhibition assay	room temperature	7.4
1JPS_HL:T	Fab D3H44	Tissue factor	11307801	1.00E-10	SPR	room temperature	7.2
1JTG_B:A	beta-lactamase inhibitor protein	beta-lactamase TEM-1	9890878	4.00E-10	SPR	25	7.5
1JWH_CD:A	Casein kinase II beta chain	Casein kinase II alpha chain	18824508	1.26E-08	ITC	35	8.5
1K5D_AB:C	Ran GTPase	Ran GAP	14585972	3.00E-10	Stopped-flow fluorescence	20	7.5
1KAC_A:B	Adenovirus fiber knob protein	Adenovirus receptor	10684297	1.48E-08	SPR	25	7.4
1KKL_ABC:H	HPr kinase C-ter domain	HPr	12009882	4.50E-08	SPR	25	8
1KLU_AB:D	MHC class 2 HLA-DR1	Staphylococcus enterotoxin C3	10229190	4.60E-06	SPR	25	7.5
1KTZ_A:B	TGF-beta	TGF-beta receptor	16300789	2.90E-07	SPR	25	7.4
1KXP_A:D	Actin	Vitamin D binding protein	2910852	9.00E-10	Inhibition assay	25	7.4
1KXQ_H:A	Camel VHH - Pancreatic alpha-amylase	Pancreatic alpha-amylase	9649422	3.50E-09	SPR	not stated	7.4
1LFD_B:A	Ras.GNP	RalGDS Ras-interacting domain	15197281	1.94E-06	Stopped-flow fluorescence	25	7.4
1M10_A:B	Von Willebrand Factor dom. A1	Glycoprotein IB-alpha	12183630	5.80E-09	SPR	25	7.4

1MAH_A:F	Acetylcholinesterase	Fasciculin	8509385	2.50E-11	Inhibition assay	26	7.5
1MLC_AB:E	Fab44.1	HEW lysozyme	10229844	9.10E-08	SPR	25	7.4
1MQ8_A:B	ICAM-1 domain 1-2	Integrin alpha-L I domain	12526797	3.00E-06	SPR	25	not stated
1NB5_AP:I	Cathepsin H	Stefin A	8898076	6.90E-11	Inhibition assay	25	7
1NCA_HL:N	Fab - Flu virus neuraminidase N9	Flu virus neuraminidase N9	9692956	8.30E-09	Fluorescence inhibition assay	room temperature	7.2
1NSN_HL:S	Fab N10 - Staphylococcal nuclease	Staphylococcal nuclease	1704035	<1e-10	ELISA inhibiton assay	not stated	not stated
1NVU_Q:S	Ras GTPase.GTP	Son of sevenless	15507210	3.60E-06	Fluorescence anisotropy	not stated	7.5
1NVU_R:S	Ras GTPase.GTP	Son of sevenless	15507210	1.90E-06		not stated	7.5
1NW9_B:A	Capase-9	BIR3 domain of XIAP (249-354)	15485396	1.30E-08	Fluorescence inhibition assay	37	7.2
1OC0_A:B	Plasminogen activator inhibitor-1	Vitronectin Somatomedin B domain	12808446	1.00E-09	Fluorescence titration	not stated	not stated
1OPH_A:B	Alpha-1-antitrypsin	Trypsin	9012804	5.00E-09	Fluorescence inhibition assay	not stated	not stated
1P2C_AB:C	FabF10.6.6	HEW lysozyme	14988501	1.02E-10	SPR	25	not stated
1PPE_E:I	Trypsin	CMTI-1 squash inhibitor	8543044	3.00E-12	Spectrophotometric inhibition assay	22	8.3

1PVH_A:B	IL6 receptor beta chain D2-D3 domains	Leukemia inhibitory factor	14527405	8.00E-08	ITC	20	7.5
1PXV_A:C	Staphylococcus aureus cystein protease	Cystein protease inhibitor	17261086	3.10E-10	Inhibition assay	not stated	7.8
1QA9_A:B	CD2	CD58	7520278	9.00E-06	SPR	37	7.4
1R0R_E:I	Subtilisin carlsberg	OMTKY	7046785	2.94E-11	Spectrophotometry	21	8.3
1R6Q_A:C	Clp protease subunit ClpA	Clp protease adaptor protein ClpS	12426582	3.30E-07	SPR	not stated	not stated
1RLB_ABCD:E	Transthyretin	Retinol binding protein	8639713	8.00E-07	Fluorescence anisotropy	20	7.4
1RV6_VW:X	PIGF receptor binding domain	Flt1 protein domain 2	8822205	1.70E-10	Inhibition assay	37	7.2
1S1Q_A:B	UEV domain	Ubiquitin	12006492	0.000635	SPR	20	7.2
1T6B_X:Y	Anthrax protective antigen	Anthrax toxin receptor	15044490	1.70E-10	Stopped-flow fluorescence	20	8
1US7_A:B	Heat shock protein 82 N-ter domain	HSP90 co-chaperone CDC37 C-ter domain	14718169	1.46E-06	ITC	30	8
1UUG_A:B	Uracyl-DNA glycosylase	Glycosylase inhibitor	8262921	<1e-13	Stopped-flow fluorescence	25	8
1VFB_AB:C	Fv D1.3	HEW lysozyme	8302837	3.70E-09	ITC	24	7.1
1WDW_BD:A	Tryptophan synthase beta chain 1	Tryptophan synthase alpha chain	12643278	2.50E-09	ITC	50	7

1WEJ_HL:F	Fab E8	Cytochrome C	2993413	7.14E-10	Spectroscopic inhibition assay	not stated	not stated
1WQ1_R:G	Ras GTPase.GDP	Ras GAP	8262937	1.70E-05	Fluorescence	30	7.5
1XD3_A:B	UCH-L3	Ubiquitin	9485312	3.00E-07	Fluorescence spectrophotometry	25	7.5
1XQS_A:C	HspBP1	Hsp70 ATPase domain	15694338	6.50E-06	SPR	room temperature	7.4
1XU1_ABD:T	TNF domain of APRIL	TNF receptor superfamily member 13B TACI CRD2 domain	10956646	6.40E-09	SPR	not stated	7.4
1YVB_A:I	Falcipain 2	Cystatin	17502099	6.50E-09	Inhibition assay	not stated	5.5
1Z0K_A:B	Rab4A GTPase.GNP	RAB4 binding domain of Rabenosyn	16034420	7.70E-06	SPR	not stated	7.5
1ZHI_A:B	BAH domain of Orc1	Sir Orc-interaction domain	19029247	2.00E-07	ITC	23	7.2
1ZLI_A:B	Carboxypeptidase B	Tick carboxypeptidase inhibitor	15561703	1.30E-09	Inhibitor assay	23	7.5
1ZM4_A:B	Elongation factor 2	Diphtheria toxin A catalytic domain	12270928	1.30E-06	Flourescence	25	7.9
2A9K_A:B	Ral-A.GDP	Mono-ADP-ribosyltransferase C3	16177825	6.00E-08	ITC	37	7.5
2ABZ_B:E	Carboxypeptidase A1	Leech carboxypeptidase inhibitor	16126224	2.80E-09	Spectroscopic inbition assay	not stated	7.5
2AJF_A:E	Angiotensin-converting enzyme 2	SARS spike protein receptor binding domain	15791205	1.62E-08	SPR	not stated	not stated
2AQ3_A:B	TCR Vbeta8.2	SEC3	20836565	1.20E-05	ITC	not stated	7.2
2B42_A:B	Xylanase	Xylanase inhibitor	162799	1.07E-	SPR	22	5

			51	09			
2B4J_AB:C	Integrase (HIV-1)	PC4 and SFRS1 interacting protein	19801648	1.09E-08	Fluorescence inhibition assay	room temperature	7.4
2BTF_A:P	Actin	Profilin	9788869	2.30E-06	Inhibition assay	room temperature	7
2C0L_A:B	TRP region of PEX5	Sterol carrier protein 2	17157249	1.09E-07	ITC	35	7.4
2FJU_B:A	Phospholipase beta 2	Rac GTPase	12657629	5.30E-06	SPR	25	not stated
2GOX_A:B	C3D	Staphylococcus aureus Efb-C	18687868	1.40E-09	SPR	25	7.4
2HLE_A:B	Ephrin B4 receptor	Ephrin B2 ectodomain	16472751	4.00E-08	ITC	25	7.8
2HQS_A:H	TolB	Pal	17375930	2.70E-08	ITC	20	7.5
2HRK_A:B	Glutamyl-t-RNA synthetase	GU-4 nucleic binding protein	17976650	9.00E-09	SPR	25	7.4
2I25_N:L	Shark single domain antigen receptor	HEW lysozyme	16446445	1.00E-09	SPR	25	7.4
2I9B_E:A	Urokinase plasminogen activator surface receptor	Urokinase-type plasminogen activator	15003263	3.30E-10	SPR	25	7.4
2J0T_A:D	MMP1 Intersitial collagenase	Metalloproteinase inhibitor 1	12515831	4.00E-10	Fluorescence inhibition assay	37	7.5
2JEL_HL:P	Fab Jel42 - HPr	HPr	9671548	2.80E-09	Fluorescence inhibition assay	23	7.2
2MTA_HL:A	Methylamine dehydrogenase	Amicyanin	8347660	4.50E-06	Spectroscopic inhibition assay	30	7.5

2NYZ_AB:D	Viral chemokine binding p. M3	Chemokine XCL1	18070938	5.00E-10	SPR	25	7.4
2O3B_A:B	NucA nuclease	NuiA nuclease inhibitor	17138564	3.20E-12	Inhibition assay	25	7
2OOB_A:B	E3 ubiquitin-protein ligase CBL-B UBA domain	Ubiquitin	17897937	6.00E-05	ITC	19.85	7
2OOR_AB:C	NAD(P) transhydrogenase subunit alpha part 1	NAD(P) transhydrogenase subunit beta	8898902	1.55E-08	Fluorescence	25	7.2
2OUL_A:B	Falcipain 2	Chagasin	17502099	1.70E-09	Inhibition assay	not stated	5.5
2OZA_B:A	MAP kinase 14	MAP kinase-activated protein kinase 2	15287722	2.50E-09	SPR	25	8
2PCB_A:B	Cyt C peroxidase	Cytochrome C	9092837	1.00E-05	ITC	25	6
2PCC_A:B	Cyt C peroxidase	Cytochrome C, yeast	11148036	1.60E-06	ITC	25	6
2PTC_E:I	Trypsin	BPTI	5041905	6.00E-14	Inhibition assay	25	8
2SIC_E:I	Subtilisin	Streptomyces subtilisin inhibitor	32173	7.12E-11	Fluorescence titration	25	7
2SNI_E:I	Subtilisin	Chymotrypsin inhibitor 2	10065709	2.00E-12	Inhibition assay	25	8.5
2TGP_Z:I	Trypsinogen	BPTI	311834	2.40E-06	Spectroscopic inhibition assay	20	8
2UUY_A:B	Trypsin	Tryptase inhibitor from tick	17391695	5.60E-09	Inhibition assay	25	8
2VDB_A:B	Serum albumin	Peptostreptococcalalbumin-binding protein	8900134	1.50E-10	Radioligand inhibition assay	ambient	not stated
2VIR_AB:C	Fab	Flu virus hemagglutinin	946107	1.00E-	SPR	25	not stat

			7	09			ed
2VIS_AB:C	Fab	Flu virus hemagglutinin	9461077	4.00E-06	SPR	25	not stated
2WPT_A:B	Colicin E9 nuclease	Im2 immunity protein	9718299	1.50E-08	Stopped-flow fluormetry	25	7
3BP8_AB:C	Mlc transcription regulator	PTS glucose-specific enzyme EIICB	18319344	4.14E-09	SPR	not stated	8
3BZD_A:B	TCR Vbeta8.2	SEC3-1A4	20836565	9.60E-08	ITC	not stated	7.2
3CPH_G:A	Ras-related protein Sec4	Rab GDP-dissociation inhibitor	18426803	3.30E-07	ITC	25	7.5
3SGB_E:I	Streptogrisin B	Ovomucoid inhibitor third domain	3555488	1.79E-11	Spectrophotometric inhibition assay	22	8.3
4CPA_A:I	Carboxypeptidase A	Potato carboxypeptidase inhibitor	4415398	5.00E-09	Spectrophotometric inhibition assay	25	7.5

Table S3. Among groups of similar structures crystalized at different resolutions, high-resolution structures result in lower errors in predicted protein-protein affinities. We report the Spearman correlation between crystal resolution and prediction error. Protein-protein complexes were clustered at 90% sequence identity. Crystal resolution was in angstroms, and prediction error was calculated as the difference between predicted and experimental pKd. Large positive correlations indicate that crystal resolution explains a large amount of the variance in prediction error among structures of similar protein sequence, with high-resolution structures producing smaller errors in pKd.

cluster size	correlation	p-value
**8**	**0.79**	**0.03**
**7**	**0.82**	**0.03**
**5**	**0.89**	**0.04**
6	-0.77	0.10
7	-0.57	0.20
5	0.67	0.22
17	-0.31	0.23
6	-0.60	0.24
4	0.74	0.26
5	-0.62	0.27
4	0.80	0.33
5	0.53	0.36
7	-0.31	0.50
6	0.20	0.70
4	-0.40	0.75
4	0.40	0.75
5	-0.20	0.78
4	-0.11	0.89
4	0.11	0.89
4	0.20	0.92
4	0.20	0.92

Table S4. Worst 10 predictions found using the Protein Affinity Benchmark and possible database errors affecting the prediction accuracy.

PDB ID	Experimental pKd	Predicted pKd	Prediction Error	Possible database errors
1EMV	13.62	8.70	4.92	Ion dependent affinity and crystallographic cofactos
1DFJ	13.23	8.41	4.82	-
2O3B	11.49	7.06	4.44	Mutation (D121A)
1Z0K	5.11	9.14	4.03	Crystallographic cofactos
1AKJ	3.90	7.80	3.90	-
2PTC	13.22	9.34	3.88	-
1WQ1	4.77	8.62	3.85	Ion dependent affinity
1XD3	6.52	10.27	3.74	-
1BRS	12.70	9.09	3.61	-
1S1Q	3.20	6.40	3.21	-

References

1. Eccleston JF, Moore KJ, Morgan L, Skinner RH, Lowe PN: **Kinetics of interaction between normal and proline 12 Ras and the GTPase-activating proteins, p120-GAP and neurofibromin. The significance of the intrinsic GTPase rate in determining the transforming ability of ras**. *J Biol Chem* 1993, **268**(36):27012-27019.
2. Wallis R, Leung KY, Pommer AJ, Videler H, Moore GR, James R, Kleanthous C: **Protein-protein interactions in colicin E9 DNase-immunity protein complexes. 2. Cognate and noncognate interactions that span the millimolar to femtomolar affinity range**. *Biochemistry-Us* 1995, **34**(42):13751-13759.
3. Paavilainen VO, Oksanen E, Goldman A, Lappalainen P: **Structure of the actin-depolymerizing factor homology domain in complex with actin**. *The Journal of cell biology* 2008, **182**(1):51-59.
4. Chan B, Lanyi A, Song HK, Griesbach J, Simarro-Grande M, Poy F, Howie D, Sumegi J, Terhorst C, Eck MJ: **SAP couples Fyn to SLAM immune receptors**. *Nature cell biology* 2003, **5**(2):155-160.

5. Beernink PT, Hwang M, Ramirez M, Murphy MB, Doyle SA, Thelen MP: **Specificity of protein interactions mediated by BRCT domains of the XRCC1 DNA repair protein**. *J Biol Chem* 2005, **280**(34):30206-30213.
